# The Core and Seasonal Microbiota of Raw Bovine Milk in Tanker Trucks and the Impact of Transfer to a Milk Processing Facility

**DOI:** 10.1128/mBio.00836-16

**Published:** 2016-08-23

**Authors:** Mary E. Kable, Yanin Srisengfa, Miles Laird, Jose Zaragoza, Jeremy McLeod, Jessie Heidenreich, Maria L. Marco

**Affiliations:** a Department of Food Science and Technology, University of California Davis, Davis, California, USA; b Hilmar Cheese Company, Hilmar, California, USA

## Abstract

Currently, the bacterial composition of raw milk in tanker trucks and the outcomes of transfer and storage of that milk at commercial processing facilities are not well understood. We set out to identify the bacteria in raw milk collected for large-scale dairy product manufacturing. Raw bovine milk samples from 899 tanker trucks arriving at two dairy processors in San Joaquin Valley of California during three seasons (spring, summer, and fall) were analyzed by community 16S rRNA gene sequencing. This analysis revealed highly diverse bacterial populations, which exhibited seasonal differences. Raw milk collected in the spring contained the most diverse bacterial communities, with the highest total cell numbers and highest proportions being those of *Actinobacteria*. Even with this complexity, a core microbiota was present, consisting of 29 taxonomic groups and high proportions of *Streptococcus* and *Staphylococcus* and unidentified members of *Clostridiales*. Milk samples were also collected from five large-volume silos and from 13 to 25 tankers whose contents were unloaded into each of them during 2 days in the summer. Transfer of the milk to storage silos resulted in two community types. One group of silos contained a high proportion of *Streptococcus* spp. and was similar in that respect to the tankers that filled them. The community found in the other group of silos was distinct and dominated by *Acinetobacter*. Overall, despite highly diverse tanker milk community structures, distinct milk bacterial communities were selected within the processing facility environment. This knowledge can inform the development of new sanitation procedures and process controls to ensure the consistent production of safe and high-quality dairy products on a global scale.

## IMPORTANCE

Raw milk harbors diverse bacteria that are crucial determinants of the quality and safety of fluid milk and (fermented) dairy products. These bacteria enter farm milk during transport, storage, and processing. Although pathogens are destroyed by pasteurization, not all bacteria and their associated enzymes are eliminated. Our comprehensive analyses of the bacterial composition of raw milk upon arrival and shortly after storage at major dairy processors showed that the communities of milk microbiota are highly diverse. Even with these differences, there was a core microbiota that exhibited distinct seasonal trends. Remarkably, the effects of the processing facility outweighed those of the raw milk microbiome and the microbial composition changed distinctly within some but not all silos within a short time after transfer. This knowledge can be used to inform cleaning and sanitation procedures as well as to enable predictions of the microbial communities in raw milk that result in either high-quality or defective products.

## INTRODUCTION

Bacteria are essential determinants of the shelf life, organoleptic qualities, and safety of fresh and (minimally) processed foods. While the microbial ecology of fresh produce (1–4) and animal products (5–10) has been intensively studied, the influence of storage, transport, and processing facilities on the microbial composition and ecology of those products is less comprehensively understood (11–14).

Bovine milk and manufactured dairy products made from it are among the most frequently consumed foods at global scales (15). Only a small fraction of produced milk is consumed as a beverage, with much higher quantities being either fermented to create other food products (e.g., cheese, yogurt, etc.) or processed for ingredients such as whey or lactose (16). Milk is safe to consume after pasteurization but is still susceptible to microbe-induced spoilage and quality defects. In contrast, some of the surviving microorganisms in milk contributes beneficially to the organoleptic qualities of fermented dairy products. Lactic acid bacteria (LAB) are particularly important because of their positive and negative impacts on fresh and fermented dairy foods (17–20).

A wide variety of bacterial species have been detected in raw and minimally processed milk (6, 10). Dairy locations and milking practices, including housing (indoor versus outdoor) and feed and bedding type, alter the bacterial populations present on cow teats, on dust, and in air in the milking parlor and ultimately contribute to the raw milk microbiome (7, 10, 21–24). By comparison, the microbiota present in fresh milk after transport and storage is not as well understood (25–27). Increases in standard plate counts (25) and slightly higher coliform counts (27) were found upon transfer of raw milk from farm tanks to dairy processor bulk tanks. Although on-farm knowledge of raw milk microbiota is important for identification of the sources of entry, the bacterial composition of raw milk as it reaches the site of pasteurization and processing is directly relevant to the production of high-quality dairy products with a long shelf life.

California is the largest dairy producer in the United States, providing the largest amount of fluid milk (42,337 million lb per year, 20.6% of total United States milk production) (28) and the second largest quantity of cheese (189.9 million lb per year), surpassed only by Wisconsin (226.3 million lb per year) (29). However, the microbial community in raw milk samples in California just prior to use for the manufacture of dairy products has not yet, to our knowledge, been comprehensively examined. Given that both the location and transportation of raw milk can affect its microbial contents, we set out to identify the microbiota of raw milk collected for large-scale product manufacturing in California. Specifically, we measured the consistency of the milk microbiota upon arrival at two processing facilities and determined how that microbiota was affected by large-scale, short-term storage at the manufacturing facility.

## RESULTS

### Bacterial populations in raw milk in tanker trucks are highly diverse.

The bacterial diversity in raw bovine milk after bulk transport was determined for 899 tanker trucks upon arrival at two different dairy processors in the California central valley. This collection included 229 tankers filled in the fall of 2013 and another 264 and 406 tankers filled in the spring and summer of 2014, respectively. The larger set of samples collected in the summer included milk collected from two sampling dates 1 week apart. For each of those tanker truck milk samples, we obtained at least 15,000 16S rRNA gene reads by high-throughput DNA sequencing that met quality-filtering requirements for further analysis.

**FIG 1:**
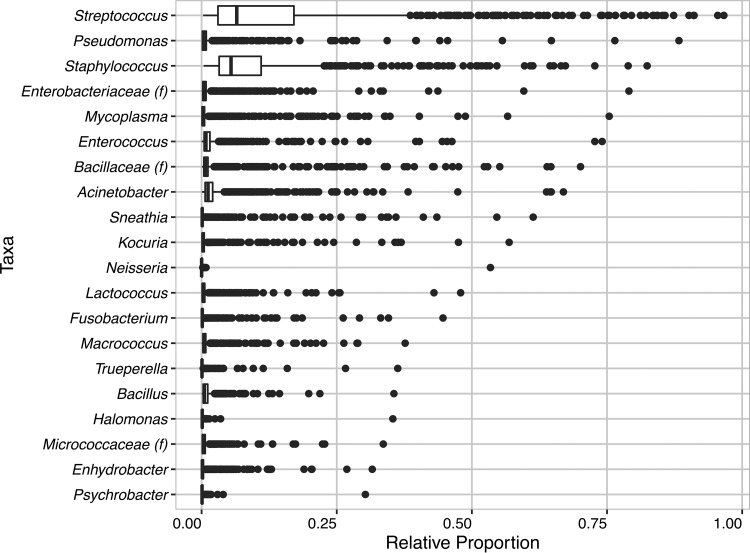
Variations in the proportions of predominant bacteria taxa in raw milk delivered to two dairy production facilities in California. Data represent relative abundances of taxa found at 30% or greater relative abundance in at least one raw tanker milk sample. Relative abundances were calculated after rarefaction of the OTU table to 15,000 sequences per sample. (f), family.

Phylogenetic and taxonomic assessments of the 16S rRNA V4 regions revealed that bacterial populations in the raw milk in the tankers were highly diverse and variable. In 354 (39.4%) of the raw milk samples analyzed, taxa detected at less than 1% relative abundance accounted for 50% or more of the bacteria present. The variation in the bacterial populations between the tankers was exemplified by the finding that while some of the trucks contained high (>30%) proportions of bacteria of certain taxa such as *Streptococcus*, *Pseudomonas*, *Staphylococcus*, and *Mycoplasma*, these same taxa were detected at very low (<1%) levels in the milk of the other tankers tested (Fig. 1).

### Core microbiome of raw milk.

Despite these differences in the raw milk bacterial communities, a core milk microbiome was still present. A total of 29 taxa were detected in 100% of the raw milk samples examined (Table 1). This core microbiome encompassed members of the *Firmicutes*, *Actinobacteria*, *Bacteroidetes*, *Proteobacteria*, and *Tenericutes* phyla. The most abundant bacterial taxa were *Streptococcus*, *Staphylococcus*, and unidentified members of order *Clostridiales* and comprised medians of 6.5, 5.4, and 6.3% of the milk microbiota, respectively (Table 1). Within the order *Clostridiales*, several identifiable taxa were also present at relatively high median relative abundances, including species of the genus *Clostridium* (1.5%) and unidentified members of families *Clostridiaceae* (1.3%) and *Lachnospiraceae* (2%). Moreover, *Corynebacterium* (3.7%), *Turicibacter* (2.5%), and *Acinetobacter* (1.2%) were also abundant. Although *Mycoplasma* composed 75% of the total community in at least 1 milk sample, its median relative abundance in all milk tested was relatively low (0.26%). Notably, *Pseudomonas* was not a part of the core microbiome. Although it was present in relatively high proportions in some of the milk tested (Fig. 1), *Pseudomonas* was entirely absent from two tankers and was therefore not included in the core. A complete list of all bacterial taxa identified in the raw tanker milk samples is provided in Table S1 in the supplemental material.

**TABLE 1:** Core milk microbiota

Phylum	Class	Order	Family	Genus	Relative median % abundance ^a^
*Firmicutes*	*Bacilli*	*Bacillales*	*Staphylococcaceae*	*Staphylococcus*	5.45
				*Salinicoccus*	0.62
				*Macrococcus*	0.45
			*Bacillaceae*	Unidentified	0.68
				*Bacillus*	0.51
			*Planococcaceae*	Unidentified	1.09
		*Lactobacillales*	*Aerococcaceae*	Unidentified	0.97
			*Enterococcaceae*	*Enterococcus*	0.81
			*Streptococcaceae*	*Streptococcus*	6.51
		*Turicibacterales*	*Turicibacteraceae*	*Turicibacter*	2.45
	*Clostridia*	*Clostridiales*	*Lachnospiraceae*	Unidentified	2.03
				*Butyrivibrio*	0.79
				*Dorea*	0.67
				*Coprococcus*	0.36
			*Clostridiaceae*	*Clostridium*	1.47
				Unidentified	1.33
			*Ruminococcaceae*	Unidentified	4.35
				*Ruminococcus*	0.84
			*Peptostreptococcaceae*	Unidentified	2.22
			Unidentified	Unidentified	6.33
*Actinobacteria*	*Actinobacteria*	*Actinomycetales*	*Micrococcaceae*	Unidentified	0.31
				*Kocuria*	0.25
			*Corynebacteriaceae*	*Corynebacterium*	3.70
			*Yaniellaceae*	*Yaniella*	0.49
*Bacteroidetes*	*Bacteroidia*	*Bacteroidales*	Unidentified	Unidentified	0.86
			*Bacteroidaceae*	*5-7N15*	0.81
*Proteobacteria*	*Gammaproteobacteria*	*Enterobacteriales*	*Enterobacteriaceae*	Unidentified	0.40
		*Pseudomonadales*	*Moraxellaceae*	*Acinetobacter*	1.19
*Tenericutes*	*Mollicutes*	*Mycoplasmatales*	*Mycoplasmataceae*	*Mycoplasma*	0.26

a All 899 raw milk samples from tanker trucks, including milk tested from three seasons, were used to determine the values.

### The microbiota in raw milk varies depending on the season.

**FIG 2:**
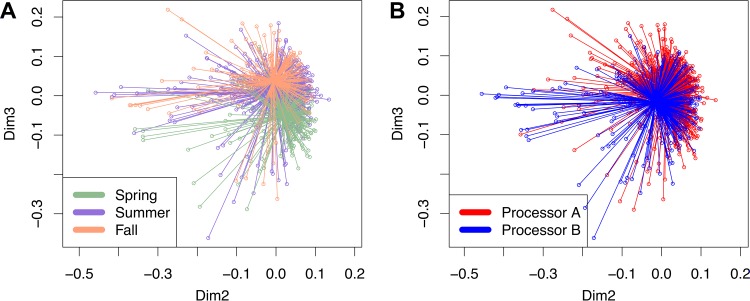
PCoA of the weighted UniFrac distances between bacterial communities in raw milk tankers. Rarefaction at 15,000 sequences per sample preceded UniFrac analysis. Milk samples are colored by (A) season (spring = light green, summer = purple, and fall = orange) and (B) processor (A = red and B = blue). Dimension 2 (Dim2) and dimension 3 (Dim3) show changes in the overall community composition between seasons but not between processors.

Because 16S rRNA gene sequencing can result in nonuniform sample coverage, operational taxonomic unit (OTU) count normalization methods are necessary prior to any comparative analyses (30, 31). Therefore, three methods for normalizing OTU counts were employed: cumulative sum scaling (CSS), CSS followed by batch correction, and rarefaction at a depth of 15,000 sequences per sample. Principal coordinate analysis (PCoA) of the weighted UniFrac distance (beta diversity) among the bacteria in the tanker milk samples was then performed on the normalized data. These comparisons showed that all three methods resulted in PCoA values with substantial overlap among all milk collected but also with some clustering according to season (Fig. 2; see also Fig. S1 in the supplemental material).

**FIG 3:**
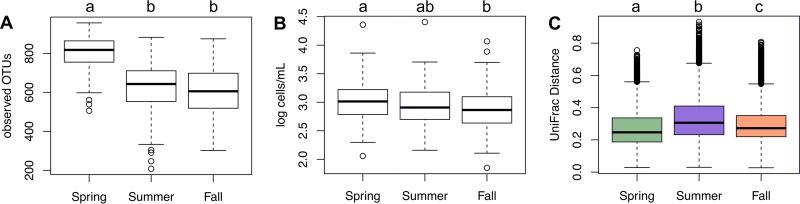
Seasonal differences in alpha (within-sample) and beta (between-sample) diversities of raw tanker milk microbial communities. Significant differences are indicated by the presence of different lowercase letters above each box plot. (A) The number of observed OTUs at 15,000 sequences per sample was significantly higher in spring than in summer or fall by the Kruskal-Wallis test (*P* value, <2.2e-16) followed by Nemenyi test pairwise comparisons (Benjamini-Hochberg-corrected *P* values, <0.001 [spring versus fall], <0.001 [spring versus summer], and not significant [ns; fall versus summer]). (B) The numbers of cells per milliliter estimated by qPCR for 365 milk samples total (134 from spring, 97 from summer, and 134 from fall). Total cell numbers differ between milk samples collected in the fall and spring by the Kruskal-Wallis test (*P* value, 0.001929) followed by Nemenyi test pairwise comparisons (Benjamini-Hochberg-corrected *P* value, 0.0012 [spring versus fall], ns [spring versus summer], and ns [summer versus fall])**.** (C) Weighted UniFrac distances among raw milk communities within each season. The distances between milk samples in spring were the lowest and in summer were the highest among the seasons tested (Kruskal-Wallis *P* value, <2.2e-16; all Nemenyi test pairwise comparison Benjamini-Hochberg-corrected *P* values were <0.001).

According to Adonis, a part of the vegan R package wrapped in QIIME, the season when the milk was collected explained about 5% of the variation in bacterial diversity between the raw milk samples (*R*^2^ = 0.04619 [rarefaction], 0.14313 [CSS], and 0.07911 [CSS with batch correction]; *P* = 0.0001). These differences were likely not due to different sampling dates alone because milk samples collected on 2 different days within the summer season were highly similar. Although alpha diversity explained 9% of the bacterial variation (*R*^2^ = 0.09199 [rarefaction], 0.05538 [CSS], and 0.07341 [CSS with batch correction]; *P* = 0.0001), this variation corresponded well to seasonal changes in the microbiota (Fig. 3A; see also Fig. S2 in the supplemental material). By comparison, sequencing depth exerted little or no influence on the variation in bacterial diversity between the samples (*R*^2^ = 0.01242 [rarefaction], 0.00642 [CSS], and 0.00873 [CSS with batch correction]; *P* = 0.0001). Similarly, the dairy processor where the milk was delivered had very little impact on the microbial composition (*R*^2^ = 0.01052 [rarefaction], 0.0164 [CSS], and 0.01332 [CSS with batch correction]; *P* = 0.0001) (Fig. 2B; see also Fig. S1 in the supplemental material).

The total estimated bacterial richness per sample differed between seasons. Raw milk collected in the spring contained the highest median species richness according to the breakaway package in R (see Fig. S2 in the supplemental material). Similarly, the highest number of OTUs was observed in milk examined in the spring (Fig. 3A). The lowest numbers of OTUs were detected in the fall. These differences also corresponded to the total estimated cell numbers (Fig. 3B). Quantitative PCR (qPCR) applied to the enumeration of bacteria in the raw milk indicated that all tankers contained an average of 1.4 × 10^3^ bacterial cells/ml. Although milk sampled in the spring contained only modestly higher numbers of cells (average of 1.6 × 10^3^ bacterial cells/ml), this difference was significant in comparison to the levels observed in the fall (Fig. 3B).

Even though bacterial species richness and median observed numbers of OTUs were highest in milk collected in the spring, there were no OTUs that were uniquely present in those milk samples and absent in the other seasons. Seven OTUs were absent from all milk collected in the spring season that were found in the summer or fall. By comparison, only two OTUs were absent from all milk sampled in the summer season and one OTU was not found in milk collected in the fall. Moreover, milk transported in different tankers in the spring season contained bacterial communities that were more similar to each other than was the case with those found in milk samples from the other two seasons (Fig. 3C). Therefore, although milk examined in the spring contained a high number of OTUs, the whole group of 264 milk samples in spring had fewer total unique OTUs than those collected in the other two seasons.

**FIG 4:**
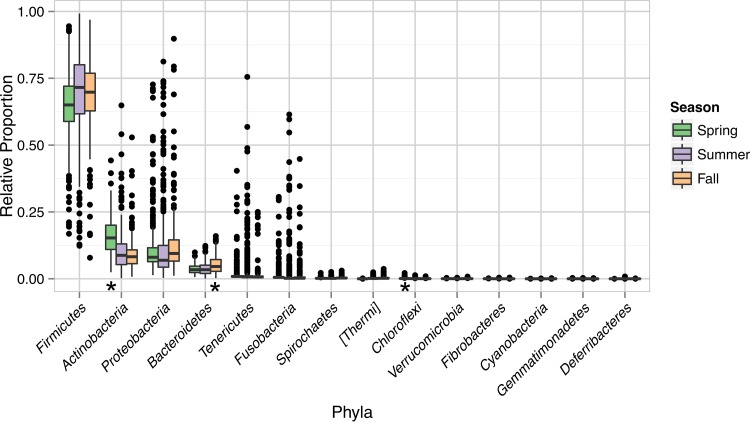
Relative proportions of the bacterial phyla present in raw tanker milk. The relative proportions of OTU counts rarefied at 15,000 sequences per sample are shown. A star beneath a box plot indicates a significant positive correlation by analysis of rarefied data with MaAslin (correlation coefficient, >0; *q* value, <0.05) and CSS-normalized data with LefSe (LDA effect size, >2; *P* value, <0.05). “*Thermi*” is a taxonomic assignment based on genome trees and is not officially recognized (i.e., Bergey's Manual of Systematic Bacteriology).

**FIG 5:**
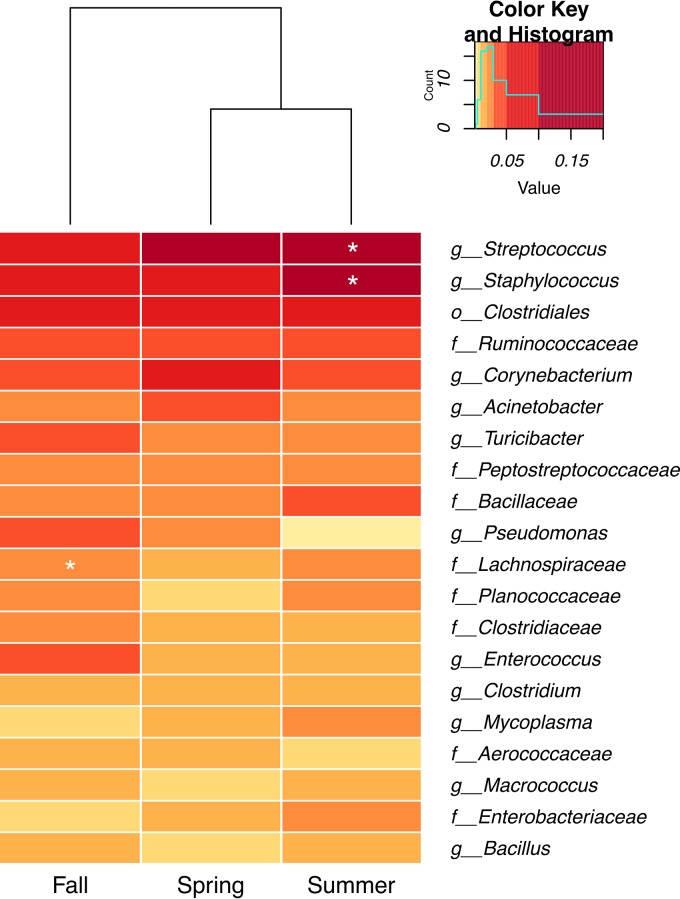
Seasonal variation in the relative proportions of abundant taxa in the raw tanker milk microbiota. Taxa present in at least 1% median relative abundance after rarefaction at 15,000 sequences per sample are shown. Those that were positively correlated with a particular season by MaAslin analysis of rarefied data (coefficient, >0; *q* value, <0.05) and LefSe analysis of CSS-normalized data (LDA effect size, > 0; *P* value, <0.05) are marked with a white star.

Consistent with the variations in alpha diversity, the relative abundances of individual bacterial taxa were dependent on the season examined. For example, *Firmicutes* species were most abundant overall but were present at significantly lower quantities in the spring than in the other seasons (Fig. 4). In contrast, the proportions of species of the *Actinobacteria* and *Chloroflexi* phyla were highest in spring (significant associations determined by both MaAsLin and LefSe), and *Bacteroidetes* numbers were significantly enriched in the fall (Fig. 4). At deeper taxonomic levels, species of the genera *Streptococcus* and *Staphylococcus* were highest in number in the summer and the populations of unidentified members of the *Lachnospiraceae* family were increased in fall (significant differences determined by both MaAsLin and LefSe) (Fig. 5).

### Impact of large-volume storage on raw milk microbial communities.

To measure whether the raw milk microbiota was retained upon transfer of milk from the tanker truck to short-term, large-volume storage, we examined milk contained in five large-volume silos (silos A, B, C, D, and E) and the tankers that filled them. These samples were collected on 2 days, 1 week apart, in the summer of 2014. Each silo was tested at least once per week except for silo D, which was tested only in the second week.

**FIG 6:**
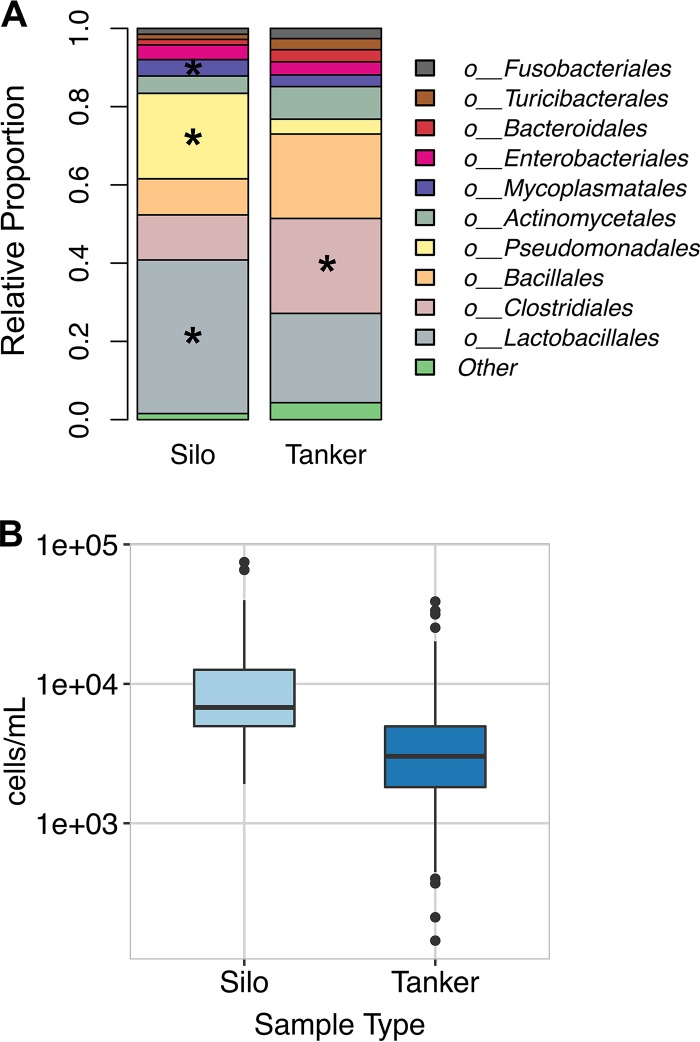
Bacterial community composition and cell number estimates for silos and tankers. (A) Relative proportions of bacterial taxa that were present in at least 2% relative abundance in silos and tankers at a rarefaction depth of 15,000 sequences. All bacterial taxa present at less than 2% relative abundance were grouped into the “other” classification. Significant positive associations are marked with a star. (B) Bacterial cell counts estimated by qPCR are shown for large-volume silos and the tankers that filled them. There is a trend for increased numbers of cells per milliliter in large-volume silos relative to tankers which approaches but does not quite reach significance (Welch’s *t* test *P* value, 0.06762).

The bacterial composition of raw milk in silos was distinct from that in the tankers, accounting for 5% of the variation by PCoA of the weighted UniFrac distances (Adonis *R*^2^ = 0.05147 [rarefied] and 0.0457 [CSS]) (see Fig. S3 in the supplemental material). This difference was consistent with increased proportions of several taxa in the silos. At the order level, *Lactobacillales* and *Pseudomonadales* numbers were significantly enriched in silos relative to tankers (Fig. 6A). *Streptococcaceae* (order *Lactobacillales*) numbers were significantly enriched in silos (statistically significant by MaAsLin and LefSe) (Fig. 6A). The numbers of organisms of the genera *Lactococcus* and *Streptococcus* within this family tended to be higher in silos than in tankers, but genus-level differences were not significant by both statistical methods used. Within the *Pseudomonadales*, the genera *Acinetobacter* and *Pseudomonas* also tended to be more abundant in silos than in tankers. Lastly, numbers of *Mycoplasma* (order *Mycoplasmatales*), a member of the core milk community, were also enriched in silos (statistically significant by MaAsLin and LefSe) (Fig. 6A). In contrast, members of the order *Clostridiales* and genus *Corynebacterium* (order *Actinomycetales*) were detected in significantly lower proportions in silos than in tankers (statistically significant by MaAsLin and LefSe) (Fig. 6A). These changes are notable because the cell density of bacteria in the silos was higher than in the tankers that filled them (Fig. 6B), suggesting that the increased relative abundances of members of *Lactobacillales* and *Pseudomonadales* orders were due to bacterial growth.

**FIG 7:**
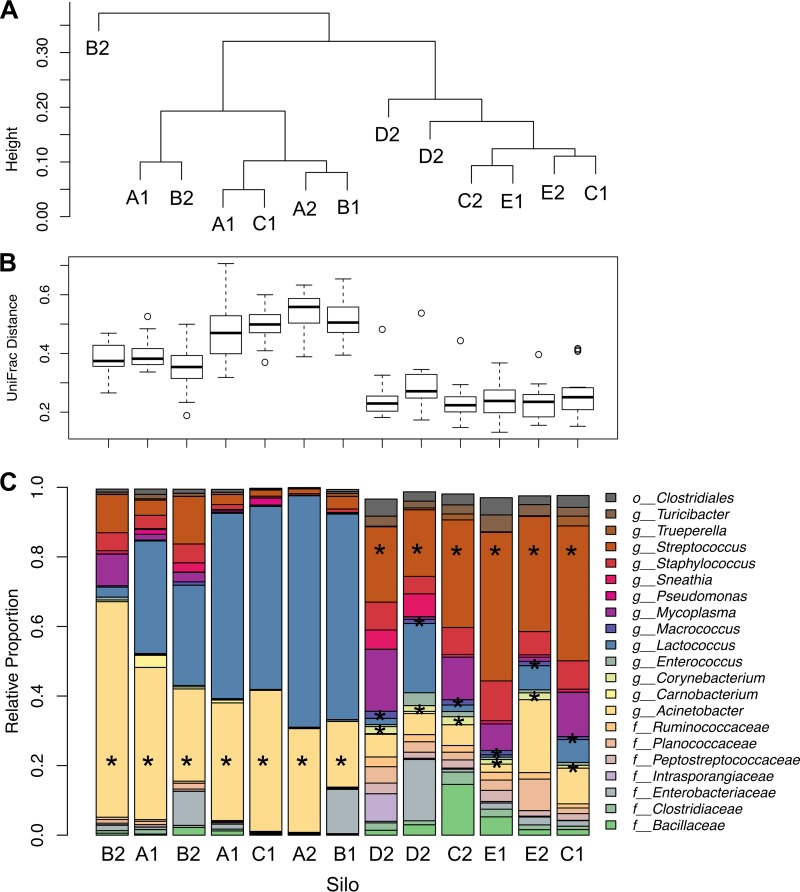
Variations in silo microbiota. (A) UPGMA cluster dendrogram of weighted UniFrac distances between raw milk silo communities. (B) Box plot of the weighted UniFrac distances between each silo on the *x* axis and the tankers that filled it. (C) Relative proportions of the taxa that were present in at least 2% relative abundance in the silo milk samples. Silos are labeled with a letter designating a physical silo and a number indicating either the first or second sampling week. Silo samples with the same designation (e.g., A1) constitute the same silo tested at different times on the same day. All analyses were performed using an OTU table that was rarefied to 15,000 sequences per sample. Significantly enriched taxa are indicated with a star.

**FIG 8:**
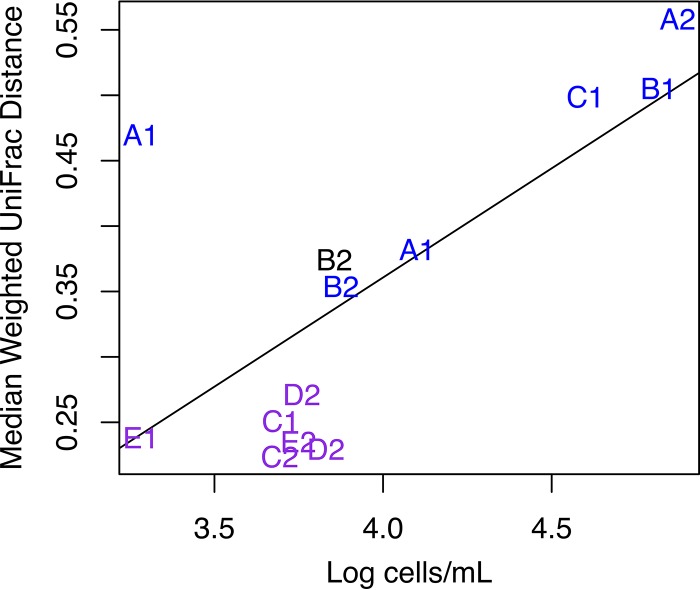
Median weighted UniFrac distances between silos and tankers compared to bacterial load. The median weighted UniFrac distances between silos and the tankers that filled them are shown on the *y* axis. The log_10_ values corresponding to the number of cells per milliliter estimated for each silo using qPCR are shown on the *x* axis. These values are linearly correlated (Pearson’s product-moment correlation, 0.706; *P* value, 0.007). Points are depicted as text with a letter designating a physical silo and a number indicating either the first or second sampling week. The text is colored by UPGMA cluster (1 = black, 2 = blue, 3 = purple). The tendency toward both higher bacterial load and larger UniFrac distance tends to be silo specific. For example, silo A has the greatest distance from corresponding tankers and E has the smallest distance, regardless of time since CIP or collection date.

Notably, not all of the bacterial communities in the silos were equally distinct from those in the tankers. Analysis of the weighted UniFrac distances for silos alone by the unweighted pair group method using average linkages (UPGMA) showed two major groups of silo-associated microbiota (Fig. 7A; see also Fig. S4A in the supplemental material). The first group included silos with bacteria that were significantly different from those in the tankers that filled them as shown by a relatively large UniFrac distance between the tankers and the companion silo (Fig. 7B; see also Fig. S4B). *Acinetobacter* numbers were significantly enriched in this group relative to the second group (statistically significant by MaAsLin and LefSe). Similarly, silo B2, an outlier according to UPGMA clustering, was also dominated by *Acinetobacter*. The second group of silo-associated bacterial communities was more similar to those in the companion tankers by weighted UniFrac and was enriched in numbers of *Streptococcus*, *Macrococcus*, and *Corynebacterium* (statistically significant by MaAsLin and LefSe) than to those in the first group. *Clostridium* was also more abundant in the second group of silos than in the first, although this genus was present at less than 2% relative abundance in the data set (data not shown). No metadata collected at the time of sampling, including clean-in-place (CIP) times and the date of sample collection, were significantly correlated to these results. However, silos A and B were present only in the first group whereas silos D and E were present only in the second. Silo C was present in both clusters. The median weighted UniFrac distance from tankers that filled each silo was significantly correlated with the number of bacterial cells per milliliter estimated for each silo (Pearson’s product-moment correlation, 0.706; *P* value, 0.007) (Fig. 8).

## DISCUSSION

We found that the bacterial populations in raw milk arriving at dairy processing facilities are highly diverse and differ according to season; however, they still contain a core, reproducible microbiota. This core microbiota was vulnerable to modification as shown by the significant change in some bacterial populations upon transfer of the milk to storage silos.

Although a number of studies have identified the bacteria in raw milk on farms and within creameries, the microbial content of milk as it reaches the site of processing has remained largely unknown. Here we found that bacterial populations in tanker milk are highly diverse and heterogeneous, with a large proportion of taxa present at less than 1% relative abundance. This finding is consistent with previous observations of raw milk in general (7). Notably, the high level of bacterial diversity in raw milk is distinct from the bacterial composition of other host-associated environments. For example, 20% or less of human fecal communities is composed of taxa below 1% relative abundance (unpublished data from our laboratory and from the American Gut Project [round 20] and data from Turnbaugh et al. [32] analyzed in QIITA; https://qiita.ucsd.edu/). Such heterogeneity might be a result of the highly nutritive content of milk and the numerous potential sources of bacteria such as aerosols, cattle skin, bedding, feed, human handling, the microbiota resident on milking equipment, on-site bulk tanks used for storage, and tanker trucks used for transport. Notably, it was previously concluded that tanker hauling and cleaning practices do not significantly impact total bacterial or thermophilic spore counts in milk (33). Therefore, the walls of the transport tankers were not likely the primary source of raw-milk-associated bacteria examined here.

There is a possibility that dead cells could have contributed to the bacterial heterogeneity of the milk examined. For the purpose of distinguishing living and dead cells, several studies have applied propidium monoazide (PMA) to measure the living fraction of cells in processed or pasteurized milk (5, 34, 35). However, because raw milk has not undergone thermal processing and therefore likely contains a preponderance of living cells, we did not use PMA treatment. We expect that any dead cells would not be consistently present and, when present, would be in such low proportions that they would not be represented among the core microbiota.

Despite the diversity and heterogeneity of bacteria in milk, consistent trends were also detected among the microbial communities according to various metrics (PCoA of the weighted UniFrac distance metric, alpha-diversity metrics, and relative abundances of specific taxa). Among the parameters examined, the season in which the milk was collected best distinguished between the microbiota and had a greater impact than processing facility, sequencing depth, or day of sampling. Seasonal variation in bacterial community composition has been observed for numerous agricultural products, including, but not limited to, wheat, lettuce, radish sprouts, and milk (2, 7, 36, 37). A dominant seasonal difference found here was that the milk samples collected in the spring contained the highest median richness in bacterial diversity. In addition to the increased diversity, the total bacterial cell counts were modestly but still significantly higher in the spring. Although there are myriad possible explanations for the increased species richness, this finding coincided with a significant increase in the relative proportion of bacteria in the phylum *Actinobacteria*. Because the *Actinobacteria* species identified here are associated with soil (38–41), it is possible that the increase in species richness observed in spring was due to seasonal changes, such as increased precipitation, promoting growth of *Actinobacteria* in the soil and transfer to the cow udder. Notably, these results were not necessarily due to increased access to the pasture in the spring, as pasture rates are low for the California Central Valley (data not shown).

Although the bacterial populations in each of the milk samples collected in the spring season were more diverse than those in the milk sampled in other seasons, they were also the most similar to each other and contained fewer unique OTUs as a group than was the case for the milk from either the summer or fall. By comparison, the highest number of unique OTUs was found in the fall. The weighted UniFrac distances between samples were also significantly greater in the fall than in the other seasons. This difference in UniFrac distances might indicate that milk collected from different dairies in the fall were exposed to a greater diversity of bacteria, possibly due to differences in feeding and housing throughout the region during that time of year. Another possibility is that the milk was undersampled and that DNA sequence analysis did not fully measure the distributions of rare taxa. Subsequent studies could therefore aim for larger sample sizes and greater sequencing depths to ensure bacterial recovery and identification.

Regardless of the sample-to-sample variation and seasonal differences, certain taxa were represented in all tanker milk examined. The core microbiota encompassed taxa from 5 phyla and 18 families. Among these bacteria, *Staphylococcus* was particularly dominant. This genus was previously detected in bovine milk and described as one of the most abundant genera in human breast milk (42–44). Both *Staphylococcus* and *Streptococcus* were detected at the highest levels in milk overall, and the populations of both were significantly enriched in the summer season. This result is in agreement with culture-based studies that have similarly found high numbers of *Streptococcus* in either the summer or winter (45–47).

Endospore-forming bacteria, including *Bacillus* and *Clostridium*, were also members of the core raw milk microbiota. These taxa are common on the dairy farm and are known to be associated with dairy processing environments (10, 48, 49). *Bacillus* and *Clostridium* also encompass species associated with spoilage of pasteurized milk and milk products. *Clostridium* species, especially *Clostridium tyrobutyricum*, are known to cause late blowing defects in hard and semihard cheeses (50). *Bacillus* species cause texture defects and off flavors in pasteurized and refrigerated fluid milk products (51). *Clostridium*, unidentified members of *Clostridiales*, and unidentified members of *Bacillaceae* tended to be present at lowest relative abundance in the summer. The lower proportion of these endospore-forming bacteria contradicts previous studies showing that the prevalence of spore formers in raw milk is high in the summer (52, 53). Notably, those studies were performed in locations with higher relative humidities than the Central Valley, CA, which has a Mediterranean or semiarid climate (54). Moreover, feeding practices differ depending on geographic location and silage in particular can serve as a source of thermoduric bacteria and endospore former contamination (48, 52, 53, 55).

*Corynebacterium* and *Acinetobacter* and taxa in the *Enterobacteriaceae* family were also members of the core microbiota and are bacteria frequently detected in raw milk (7). Of these genera, *Corynebacterium* is regarded as both thermoduric and psychotropic (56). Members of this genus contribute beneficially to flavor development of smear-ripened cheeses (57). Although *Corynebacterium* was found in all tankers examined, numbers of this member of the *Actinobacteria* phylum tended to be enriched in spring, suggesting that product flavor outcomes might be impacted by seasonal changes in the raw milk microbial community. In contrast, *Gammaproteobacteria* such as both *Acinetobacter* and *Enterobacteriaceae* are associated with spoilage (58). *Acinetobacter* is also a psychrotroph; however, it is generally unable to survive pasteurization (59). Notably, *Acinetobacter* and *Enterobacteriaceae* tended to be more abundant in the spring and summer, whereas *Pseudomonas*, another member of the *Gammaproteobacteria*, was more prevalent in the fall. Overall, these results indicate a seasonal component with respect to the dominant spoilage and nonstarter bacteria in milk. Although pasteurization eliminates the majority of impacts of these organisms on dairy products, these bacteria and their cellular components, including heat-stable extracellular proteases and lipases (60), can sometimes survive thermal treatment or reenter through processing lines (5, 59, 61–63). The metabolic and stress tolerance distinctions between different bacterial species could provide new opportunities to develop different hygiene measures targeting the most problematic (or desirable) species on a seasonal basis.

Lastly, *Streptococcus* was found in the highest relative abundance among all the identified genera and was a member of the core milk microbiota. Both *Streptococcus* and *Enterococcus*, a LAB relative of *Streptococcus* and member of the core microbiota, were previously shown to be among the most abundant LAB in raw bovine milk collected directly from dairy farms (10) and dairy production facilities (5, 7, 64, 65). Compared to other LAB genera, *Streptococcus* and *Enterococcus* are recognized to be highly thermoduric (66) and therefore might survive pasteurization. Both *Streptococcus* and *Enterococcus* are typically detected together with other LAB such as *Lactococcus*, *Lactobacillus*, and *Leuconostoc* in raw milk (6, 7). Taken together, these bacteria are generally important in fermented dairy product processing because they can significantly alter early acidification stages of cheese ripening and flavor development (67–70). Notably, *Lactococcus*, *Lactobacillus*, and *Leuconostoc* were present at very low median relative abundances of 0.27%, 0.15%, and 0.03%, respectively, in our raw milk samples and were not present in the core. Although the reasons for this are not clear, it is notable that *Lactococcus* was present at high relative proportions in some of the milk contained in large-volume holding silos (Fig. 7), suggesting that these LAB might grow from low starting quantities in milk at the time of collection.

By comparing the bacterial populations in milk in tanker trucks and silos examined in the summer season, we established that there was a rapid transformation in the microbial composition in milk upon entry into dairy processing facilities. Milk from the silos contained significant increases in the relative abundances of *Lactobacillales* and *Pseudomonadales* compared to the subset of tankers that filled them. Within these phyla, bacteria such as *Lactococcus*, *Streptococcus*, *Acinetobacter*, and *Pseudomonas* tended to be present in relatively higher proportions in the silos than in the tanker trucks. It is technically possible that some of this difference could be due to incomplete mixing within the silos. However, mixing practices were the same for all silos and the enrichments were not evenly distributed throughout all silos. Approximately half of the silos contained bacterial populations that were very similar to those in the tankers used to fill them. These silos tended to have a higher relative abundance of *Streptococcus*. The other silos were populated with a microbiota that was clearly distinct and separate from that in the tankers. These silos contained greater proportions of *Acinetobacter* and *Lactococcus*. Moreover, although *Lactococcus* can be used as a starter culture in cheese fermentations, because this organism was not consistently enriched in the silos, the recovery of this organism was likely not the result of cross-contamination due to aerosols or other transfer mechanisms on site. Our findings, in combination with increased bacterial cell count estimates within those silos, suggest that members of those species grew during cold storage.

Similarly to our findings, bacterial numbers were previously found to increase during low-temperature containment (25). The relative proportions of *Acinetobacter* were also found to increase (71). The notion that these specific genera were enriched by cold storage is supported by the fact that species of *Acinetobacter*, *Lactococcus*, and *Streptococcus* are regarded to be psychrotrophs (72, 73). However, the development of two distinct silo communities indicates that processes other than just cold storage might have determined the community development. No taxa were significantly associated with tankers that filled a given set of silos, indicating that tanker communities are not a strong predictor of silo community composition. Equipment-associated, persistent biofilms or other external contamination sources might instead predispose individual silos toward specific microbial community structures. Regardless, the observed differences between bacterial populations before and after transfer and short-term (3-to-6-h) storage in different containment vessels show that these populations are dynamic and can change quickly in response to new conditions within food processing facilities.

In conclusion, we have shown that the raw milk microbial communities that we examined were similar to each other despite being collected from different farms, transported to different locations, and sampled at different times of the year. Beyond the core milk microbiota in California, the bacterial content changed depending on season and containment equipment (truck or silo). Importantly, the bacterial composition of raw milk can be dramatically, yet variably, impacted during low-temperature, short-term storage. This knowledge is crucial to identifying the bacteria that are responsible for sporadic but consistent defects in cheese and other dairy products and developing improved methods for the treatment and handling of raw and processed milk to ensure the production of consistently high-quality products.

## MATERIALS AND METHODS

### Milk source and sampling.

Milk was collected with a stainless steel dipper from the inlet at the top of tanker trucks with 6,000-gal (22,712-liter) capacity upon arrival at two dairy processors on 7 October 2013 (fall), 13 October 2013 (fall), 5 March 2014 (spring), 11 March 2014 (spring), 26 August 2014 (summer), 27 August 2014 (summer), and 1 September 2014 (summer). For the summer samples, the local weather conditions on the two collection dates were very similar (80°F with no precipitation and wind speeds of 5 mph). Samples were collected from individual trucks over a 20-h period on each sampling date. After collection, milk samples were placed in sterile 50-ml tubes or 400-ml bags and kept at 4°C. At the end of the 20-h sampling period, samples were shipped overnight on ice to Davis, CA, where they were processed for bacterial DNA extraction immediately upon arrival (a total of 12 to 32 h after collection). The tankers contained milk of variable grades from between one and three different dairy farms of a total of 200 farms located in Merced, Stanislaus, Tulare, Kings, Fresno, Madera, Kern, and/or San Joaquin county in California. Dairy size and milking practices were varied, although they were consistent insofar as silage and alfalfa were the primary feed types and milk was held no longer than 48 h between pickup and delivery. Although there was not a single cleaning protocol for the tankers, valid wash tags, indicating that tankers had been washed within the previous 24 h, were checked prior to unloading at both facilities. A total of 974 raw tanker milk samples were collected, including 273 from spring (148 from processor A and 125 from processor B), 451 from summer (300 from processor A and 151 from processor B), and 244 from fall (126 from processor A and 118 from processor B). On 26 August 2014 and 1 September 2014, milk was also sampled from five large-volume-capacity silos at processor A. The silos were cleaned when empty (approximately every 48 h). They were kept at temperatures below 7.2°C, and the samples were collected from a valve located at the silo base immediately after they were filled. For each silo, 13 to 25 corresponding tanker milk trucks were measured throughout the filling period and labeled with the silo lot identifier (ID) number. Mixing within the silos was a result of the filling process and mechanical agitation. Silo fill times were variable, but filling was typically completed within 3 to 6 h.

### Bacterial genomic DNA extraction.

Bacterial cells were collected from 25 to 30 ml raw milk by centrifugation at 13,000 × *g* at 4°C for 5 min. The cells were then suspended in phosphate-buffered saline (PBS; pH = 7.2; 137 mM NaCl, 2.68 mM KCl, 10.1 mM Na_2_HPO_4_, 1.76 mM KH_2_PO_4_) and centrifuged a second time at 13,000 × *g* for 2 min. Bacterial pellets were stored at −20°C until DNA extraction. A PowerFood microbial DNA isolation kit (Mo Bio Laboratories, Inc., Carlsbad, CA) was used for DNA extraction and purification according to the manufacturer’s protocol, with the exception that instead of subjecting MicroBead tubes (Mo Bio Laboratories, Inc., Carlsbad, CA) to vortex mixing, the cell suspensions were shaken twice for 1 min at 6.5 m/s on a FastPrep-24 instrument (MP Biomedicals LLC).

### Bacterial count estimates by quantitative real-time PCR.

Bacterial cell numbers were estimated using quantitative PCR (qPCR). Each reaction mixture contained SsoFast Evagreen Supermix with Low ROX (Bio-Rad Laboratories, Inc., USA) and 400 nM UniF (5′-GTGSTGCAYGGYYGTCGTCA-3′) and UniR (5′-ACGTCRTCCMCNCCTTCCTC-3′) (74). qPCR was performed in a 7500 Fast Real Time PCR system (Applied Biosystems, Foster City, CA) with initiation at 95°C for 20 s followed by 40 cycles of 95°C for 3 s and 60°C for 30 s. Nonspecific amplification was evaluated by melting curve analysis. Bacteria were enumerated by comparisons of average threshold cycle (*C_T_*) values (*n* = 2) to a genomic DNA standard curve that was run on the same qPCR plate. The standard curve was prepared from DNA extracted from *Lactobacillus casei* BL23 grown to exponential phase (*A*_600_ = 0.5 to 0.7) at 37°C in MRS broth (Becton Dickinson and Company, Sparks, MD). *L. casei* cell numbers were determined by plating serial dilutions of the exponential-phase cells onto MRS agar for colony enumeration.

### 16S rRNA gene sequencing.

The V4 region of 16S rRNA genes was amplified with primers F515 and R806 with a random 8-bp bar code on the 5′ end of F515 (75, 76). PCR amplification was performed using Ex Taq DNA polymerase (TaKaRa, Otsu, Japan) with 35 cycles of 94°C for 45 s, 54°C for 60 s, and 72°C for 30 s. The PCR products were pooled and then purified with a Wizard SV Gel and PCR cleanup system (Promega, Madison, WI). NEXTflex adapters (Bioo Scientific, Austin, TX) were ligated to the 16S rRNA amplicons prior to 250-bp paired-end sequencing performed on an Illumina MiSeq instrument at the University of California, Davis (http://dnatech.genomecenter.ucdavis.edu/).

### 16S rRNA gene sequence analysis.

FASTQ files were analyzed using QIIME version 1.9.1 (77). Paired-end sequences were joined using fastq-join (78) with 175 bp overlap and a 1% maximum difference required between all paired sequences. The split_libraries_fastq.py script was used for demultiplexing and quality filtering of the resulting joined sequences. Demultiplexing was performed only with bar codes containing no sequencing errors, and quality filtering was performed at a Phred quality threshold of 30. Chimeric sequences were identified with USEARCH (79, 80) and removed. The remaining DNA sequences were grouped into OTUs with 97% matched sequence identity by the use of the “open reference” OTU picking method in QIIME with default settings. Greengenes 13_8 was used as the reference database (81) for both chimera checking and OTU picking. Sequences were aligned by the use of PyNAST (81, 82), and taxonomy was assigned using the taxonomy database in Greengenes (83, 84). The resulting OTU counts were filtered to remove any OTU that occurred at less than 0.005% relative abundance in the raw tanker milk data set (85). OTUs occurring at less than 0.016% in the silos-versus-tankers data set (10,521,986 sequences) and less than 0.288% in the silos-versus-silos data set (583,436 sequences) were also removed to focus our analysis on the more abundant bacteria within the processing facility.

### Statistics.

OTU counts were adjusted by rarefaction at 15,000 sequences per sample or by cumulative sum scaling (CSS) (30) in QIIME. DNA from five milk samples collected in the fall season was PCR amplified and used in each of five MiSeq runs. These samples were labeled as controls, and principal coordinate analysis in the QIIME package was used to determine the presence of a batch effect due to PCR amplification and sequencing. A confounding batch effect was present for raw tanker milk samples between sequencing runs in examinations performed using CSS normalization or unweighted UniFrac. Therefore, unweighted UniFrac values were not used in this study and batch correction was performed on CSS-normalized data using the ComBat function in the sva package in bioconductor (86, 87). Illustrations of these analyses were generated using the R vegan package (88).

The statistical significance of differences in community composition was determined by analyzing the weighted UniFrac distances with Adonis from the R vegan package wrapped in QIIME with 9,999 permutations. Prior to performing alpha diversity or differential abundance testing, the sequencing controls were removed from the OTU table after normalization (CSS with batch correction or rarefaction) was performed. This allowed correction of batch effects without skewing the differential abundance results due to the presence of replicate samples.

The alpha diversity of each sample was determined in two ways. First, the relative number of OTUs observed per sample in each season was evaluated at a sequencing depth of 15,000 by the use of the Kruskal-Wallis test followed by the Nemenyi test with the Tukey method for pairwise comparisons and *P* values were adjusted using Benjamini-Hochberg false-discovery-rate correction. Second, the breakaway Species Richness Estimation and Modeling R package (89) was used to estimate the total species richness of each sample and the results were compared in the same way as the rarefied alpha diversity values.

To determine differences in the relative abundances of specific taxonomic classifications between experimental groups, OTU counts from the rarefied OTU table were summed by the most specific taxonomic classification identified for each OTU and analyzed using MaAsLin (multivariate analysis with linear modeling) version 0.0.3. MaAsLin is a statistical software package that applies removal of low-abundance values, boosting, and a multivariate linear model followed by Bonferroni false-discovery-rate correction to identify taxa that are significantly associated with particular metadata (http://huttenhower.sph.harvard.edu/maaslin) (90, 91). The data from the CSS-normalized OTU table with batch correction were similarly summed and analyzed using LefSe (92). LefSe was selected over metagenomeSeq because the results were more conservative (30). Differences were considered significant by analysis in MaAslin if the *q* value (corrected *P* value) was less than 0.05 and in LefSe if the *P* value was less than 0.05 and the linear discriminatory analysis (LDA) effect size was greater than 2.0. Bacterial features are reported in the body of the text as differentially abundant between groups if the LefSe analysis of the CSS-normalized OTU table with batch correction agreed with the MaAsLin analysis of the rarefied OTU table results for that particular feature.

The silo milk microbial communities and those in the companion tanker milk samples were sequenced in two sequencing runs. No batch effect was detected for these two runs, and therefore, batch correction was not necessary prior to analysis of the impact of silo storage on raw milk microbial communities. In order to evaluate only the most abundant bacterial taxa in the silo communities, only the taxa present at greater that 2% relative abundance were evaluated using the statistical methods described above.

### Accession numbers.

Joined and demultiplexed DNA sequences were deposited in the Qiita database (https://qiita.ucsd.edu) under study ID 10485 and in the European Nucleotide Archive (ENA) under accession number ERP015209.

## SUPPLEMENTAL MATERIAL

Figure S1  PCoA of the weighted UniFrac distance metrics between bacterial communities from 6,000-gal (22,712 liters) raw milk tankers delivering to two dairy production facilities in California. The OTU table was normalized using CSS (top panels), CSS followed by batch correction (middle panels), and rarefaction to 15,000 sequences per sample (bottom panels) prior to UniFrac analysis. The left panels are colored by season, and the right panels are colored by processor. Dimensions 1, 2, and 3 are shown for each normalization method. Dimensions 2 and 3 show changes in the overall community composition between seasons but not between processors. Download Figure S1, TIF file, 2.7 MB

Figure S2  Seasonal differences in total estimated richness of raw tanker milk microbial communities as determined by the R package breakaway. (A) Summary of the estimated bacterial richness of each raw tanker milk sample per season. Significant seasonal differences in the bacterial richness were present as determined by the Kruskal-Wallis test (*P* value, <2.2e-16) followed by a Nemenyi test pairwise comparison (Benjamini-Hochberg-corrected *P* values, 2.595023e-07 [fall versus spring], 9.658940e-14 [fall versus summer], and <0.0000001 [summer versus spring]). (B) Raw data from the breakaway package showing the estimated bacterial richness of every raw tanker milk sample with the standard error of each prediction represented as a vertical line. Even with the error inherent in this prediction, a trend of increased bacterial diversity in spring is visible. Samples are separated on the *x* axis by company (processor B, samples 1 to 362; processor A, samples 363 to 899) and by season. Download Figure S2, TIF file, 2.6 MB

Figure S3  PCoA of the weighted UniFrac distances among raw tanker milk and silo milk communities collected in summer. OTU counts were normalized by rarefaction (A) and CSS (B). Raw tanker milk community data are colored in purple, raw silo milk community data are colored in red. A 95% confidence ellipse is depicted around each group. Download Figure S3, TIF file, 2.5 MB

Figure S4  Variation in silo microbiota. (A) UPGMA cluster dendrogram of weighted UniFrac distances between raw milk silo communities. Two groups are present. (B) Box plot of the weighted UniFrac distances between each silo on the *x* axis and the tankers that filled it. (C) Relative proportions of the taxa that were present in at least 2% relative abundance in each silo milk community. Silos are labeled with a letter designating a physical silo and a number indicating either the first or second sampling week. Silo samples with the same designation (e.g., A1) constitute the same silo sampled at a different time on the same day. All analyses were performed using an OTU table that was normalized using CSS. Download Figure S4, TIF file, 2.7 MB

Table S1  All bacterial taxa observed in raw tanker milk. Taxa present after rarefaction at 15,000 sequences per sample are listed with median relative abundance values. Table S1, PDF file, 0.2 MB
