# Supplementary figures and images for: The Core and Seasonal Microbiota of Raw Bovine Milk in Tanker Trucks and the Impact of Transfer to a Milk Processing Facility
Source: mBio. 2016 Aug 23;7(4):e00836-16. doi: 10.1128/mBio.00836-16 (PMC4999540; doi:10.1128/mBio.00836-16)

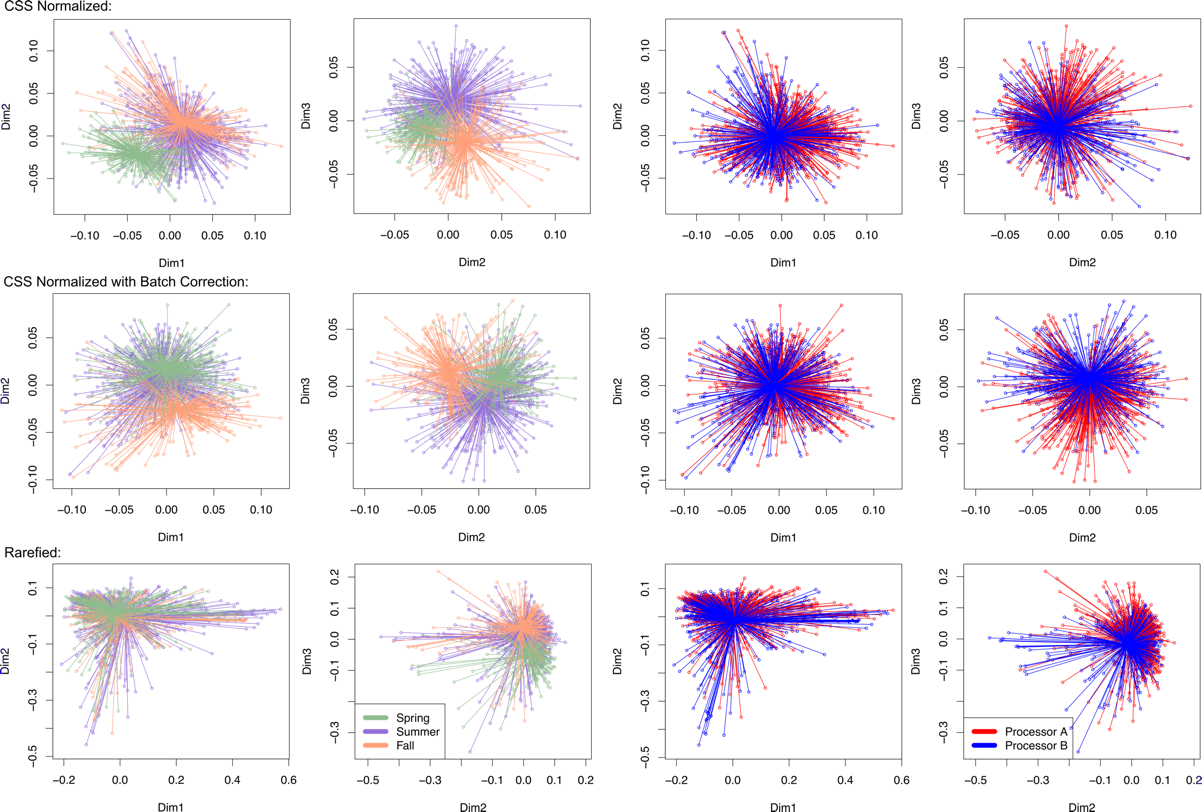

Supplement: Figure S1 — PCoA of the weighted UniFrac distance metrics between bacterial communities from 6,000-gal (22,712 liters) raw milk tankers delivering to two dairy production facilities in California. The OTU table was normalized using CSS (top panels), CSS followed by batch correction (middle panels), and rarefaction to 15,000 sequences per sample (bottom panels) prior to UniFrac analysis. The left panels are colored by season, and the right panels are colored by processor. Dimensions 1, 2, and 3 are shown for each normalization method. Dimensions 2 and 3 show changes in the overall community composition between seasons but not between processors. Download [file mbo004162952sf1.tif]

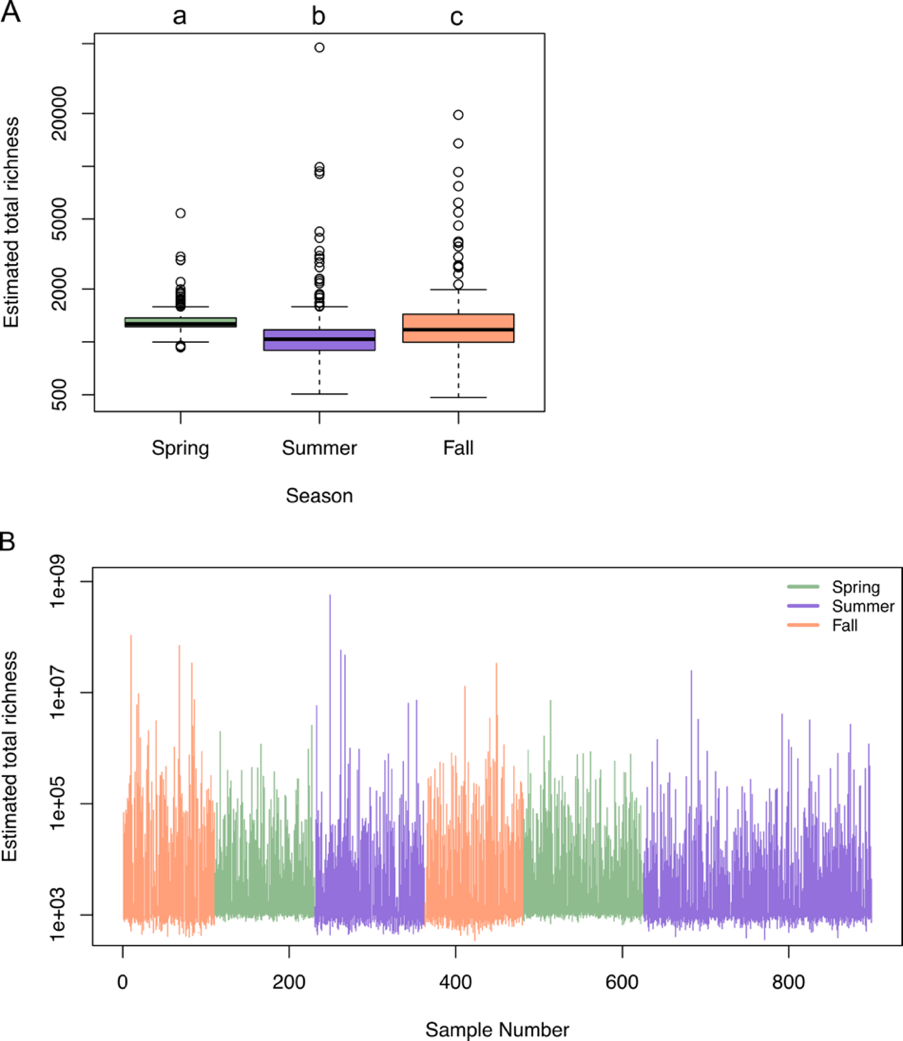

Supplement: Figure S2 — Seasonal differences in total estimated richness of raw tanker milk microbial communities as determined by the R package breakaway. (A) Summary of the estimated bacterial richness of each raw tanker milk sample per season. Significant seasonal differences in the bacterial richness were present as determined by the Kruskal-Wallis test (P value, <2.2e-16) followed by a Nemenyi test pairwise comparison (Benjamini-Hochberg-corrected P values, 2.595023e-07 [fall versus spring], 9.658940e-14 [fall versus summer], and <0.0000001 [summer versus spring]). (B) Raw data from the breakaway package showing the estimated bacterial richness of every raw tanker milk sample with the standard error of each prediction represented as a vertical line. Even with the error inherent in this prediction, a trend of increased bacterial diversity in spring is visible. Samples are separated on the x axis by company (processor B, samples 1 to 362; processor A, samples 363 to 899) and by season. Download [file mbo004162952sf2.tif]

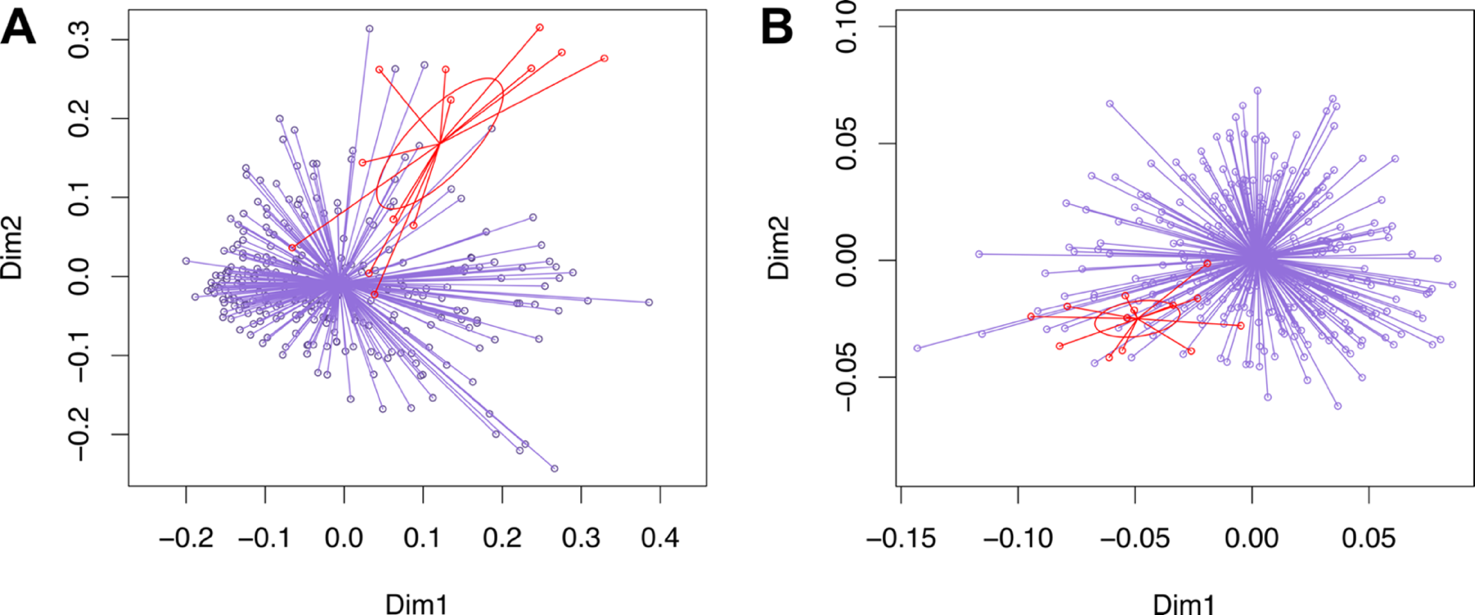

Supplement: Figure S3 — PCoA of the weighted UniFrac distances among raw tanker milk and silo milk communities collected in summer. OTU counts were normalized by rarefaction (A) and CSS (B). Raw tanker milk community data are colored in purple, raw silo milk community data are colored in red. A 95% confidence ellipse is depicted around each group. Download [file mbo004162952sf3.tif]

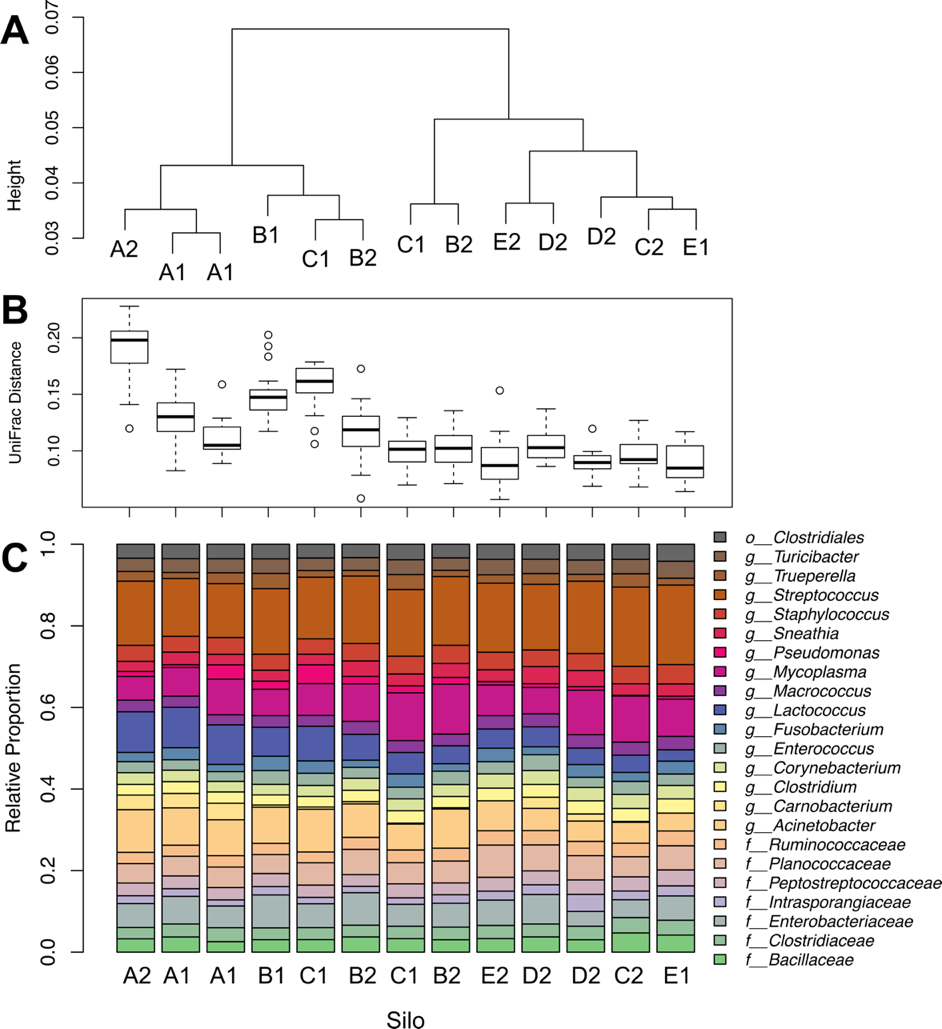

Supplement: Figure S4 — Variation in silo microbiota. (A) UPGMA cluster dendrogram of weighted UniFrac distances between raw milk silo communities. Two groups are present. (B) Box plot of the weighted UniFrac distances between each silo on the x axis and the tankers that filled it. (C) Relative proportions of the taxa that were present in at least 2% relative abundance in each silo milk community. Silos are labeled with a letter designating a physical silo and a number indicating either the first or second sampling week. Silo samples with the same designation (e.g., A1) constitute the same silo sampled at a different time on the same day. All analyses were performed using an OTU table that was normalized using CSS. Download [file mbo004162952sf4.tif]
