# Supplementary material for: The Core and Seasonal Microbiota of Raw Bovine Milk in Tanker Trucks and the Impact of Transfer to a Milk Processing Facility
Source: mBio. 2016 Aug 23;7(4):e00836-16. doi: 10.1128/mBio.00836-16 (PMC4999540; doi:10.1128/mBio.00836-16)
Supplement: Table S1 — All bacterial taxa observed in raw tanker milk. Taxa present after rarefaction at 15,000 sequences per sample are listed with median relative abundance values. [file mbo004162952st1.pdf]

Table S1. All bacterial taxa observed in raw tanker milk

Phylum	Class	Order[a]	Family[a]	Genus[a]	% median relative abundance
Firmicutes	*Bacilli*	*Lactobacillales*	*Leuconostocaceae*	*Leuconostoc*	0.026666667
Firmicutes	*Clostridia*	*Clostridiales*			6.333333333
Firmicutes	*Erysipelotrichi*	*Erysipelotrichales*	*Erysipelotrichaceae*		0.213333333
Firmicutes	*Clostridia*	*Clostridiales*	*Lachnospiraceae*	*Coprococcus*	0.36
Firmicutes	*Clostridia*	*Clostridiales*	*Clostridiaceae*		1.333333333
Firmicutes	*Bacilli*	*Bacillales*	*Staphylococcaceae*	*Staphylococcus*	5.446666667
Firmicutes	*Clostridia*	*Clostridiales*	*Lachnospiraceae*		2.026666667
Firmicutes	*Bacilli*	*Lactobacillales*	*Lactobacillaceae*	*Lactobacillus*	0.146666667
Firmicutes	*Bacilli*	*Bacillales*	*Planococcaceae*		1.093333333
Firmicutes	*Erysipelotrichi*	*Erysipelotrichales*	*Erysipelotrichaceae*	*Bulleidia*	0.006666667
Firmicutes	*Bacilli*	*Lactobacillales*	*Carnobacteriaceae*	*Trichococcus*	0.24
Firmicutes	*Clostridia*	*Clostridiales*	*[Mogibacteriaceae]*	*Mogibacterium*	0.113333333
Firmicutes	*Bacilli*	*Lactobacillales*	*Streptococcaceae*	*Streptococcus*	6.506666667
Firmicutes	*Clostridia*	*Clostridiales*	*Ruminococcaceae*		4.353333333
Firmicutes	*Bacilli*	*Bacillales*	*Paenibacillaceae*	*Brevibacillus*	0
Firmicutes	*Bacilli*	*Lactobacillales*	*Lactobacillaceae*		0.013333333
Firmicutes	*Bacilli*	*Bacillales*	*Bacillaceae*	*Bacillus*	0.513333333
Firmicutes	*Clostridia*	*Clostridiales*	*Ruminococcaceae*	*Ruminococcus*	0.84
Firmicutes	*Bacilli*	*Bacillales*	*Staphylococcaceae*	*Salinicoccus*	0.62
Firmicutes	*Clostridia*	*Clostridiales*	*Lachnospiraceae*	*Dorea*	0.666666667
Firmicutes	*Bacilli*	*Lactobacillales*	*Aerococcaceae*		0.973333333
Firmicutes	*Bacilli*	*Lactobacillales*	*Streptococcaceae*	*Lactococcus*	0.273333333
Firmicutes	*Bacilli*	*Bacillales*	*Staphylococcaceae*	*Jeotgalicoccus*	0.373333333

Firmicutes	*Clostridia*	*Clostridiales*	*Clostridiaceae*	*Proteiniclasticum*	0.08
Firmicutes	*Bacilli*	*Lactobacillales*	*Lactobacillaceae*	*Pediococcus*	0
Firmicutes	*Clostridia*	*Clostridiales*	*[Mogibacteriaceae]*		0.366666667
Firmicutes	*Clostridia*	*Clostridiales*	*Clostridiaceae*	*SMB53*	0.013333333
Firmicutes	*Clostridia*	*Clostridiales*	*[Tissierellaceae]*	*GW-34*	0.053333333
Firmicutes	*Bacilli*	*Bacillales*	*Planococcaceae*	*Rummeliibacillus*	0.086666667
Firmicutes	*Clostridia*	*Clostridiales*	*Lachnospiraceae*	*Anaerostipes*	0.006666667
Firmicutes	*Bacilli*	*Bacillales*	*Bacillaceae*	*Virgibacillus*	0.013333333
Firmicutes	*Bacilli*	*Bacillales*	*Thermoactinomycetaceae*	*Planifilum*	0.006666667
Firmicutes	*Clostridia*	*Clostridiales*	*Ruminococcaceae*	*Oscillospira*	0.266666667
Firmicutes	*Bacilli*	*Turicibacterales*	*Turicibacteraceae*	*Turicibacter*	2.453333333
Firmicutes	*Erysipelotrichi*	*Erysipelotrichales*	*Erysipelotrichaceae*	*Sharpea*	0
Firmicutes	*Clostridia*	*Clostridiales*	*[Tissierellaceae]*	*Tissierella_Soehngenia*	0.173333333
Firmicutes	*Clostridia*	*Clostridiales*	*Peptostreptococcaceae*		2.22
Firmicutes	*Clostridia*	*Clostridiales*	*Peptococcaceae*	*rc4-4*	0.173333333
Firmicutes	*Bacilli*	*Bacillales*	*Staphylococcaceae*	*Macrococcus*	0.453333333
Firmicutes	*Bacilli*	*Bacillales*	*Bacillaceae*		0.68
Firmicutes	*Erysipelotrichi*	*Erysipelotrichales*	*Erysipelotrichaceae*	*Erysipelothrix*	0.006666667
Firmicutes	*Bacilli*	*Bacillales*	*Planococcaceae*	*Lysinibacillus*	0.14
Firmicutes	*Clostridia*	*Clostridiales*	*[Tissierellaceae]*	*Helcococcus*	0.053333333
Firmicutes	*Bacilli*	*Lactobacillales*	*Aerococcaceae*	*Facklamia*	0.5
Firmicutes	*Bacilli*	*Bacillales*	*Bacillaceae*	*Natronobacillus*	0.013333333
Firmicutes	*Bacilli*	*Bacillales*			0.326666667
Firmicutes	*Clostridia*	*Clostridiales*	*Lachnospiraceae*	*Butyrivibrio*	0.786666667
Firmicutes	*Clostridia*	*Clostridiales*	*[Tissierellaceae]*		0.04
Firmicutes	*Erysipelotrichi*	*Erysipelotrichales*	*Erysipelotrichaceae*	*Catenibacterium*	0.013333333
Firmicutes	*Bacilli*	*Lactobacillales*	*Streptococcaceae*		0.006666667

Firmicutes	*Bacilli*	*Bacillales*	*Listeriaceae*		0
Firmicutes	*Clostridia*	*Clostridiales*	*Clostridiaceae*	*Clostridium*	1.466666667
Firmicutes	*Clostridia*	*Clostridiales*	*Christensenellaceae*		0.013333333
Firmicutes	*Bacilli*	*Lactobacillales*	*Carnobacteriaceae*	*Desemzia*	0
Firmicutes	*Bacilli*	*Bacillales*	*[Exiguobacteraceae]*		0
Firmicutes	*Clostridia*	*Clostridiales*	*Lachnospiraceae*	*Roseburia*	0.026666667
Firmicutes	*Clostridia*	*Clostridiales*	*Eubacteriaceae*	*Garciella*	0.013333333
Firmicutes	*Clostridia*	*Clostridiales*	*Lachnospiraceae*	*Epulopiscium*	0.206666667
Firmicutes	*Bacilli*	*Lactobacillales*	*Enterococcaceae*	*Enterococcus*	0.806666667
Firmicutes	*Clostridia*	*Clostridiales*	*[Tissierellaceae]*	*ph2*	0
Firmicutes	*Bacilli*	*Lactobacillales*	*Leuconostocaceae*	*Weissella*	0
Firmicutes	*Clostridia*	*Clostridiales*	*Lachnospiraceae*	*Blautia*	0.24
Firmicutes	*Bacilli*	*Bacillales*	*Thermoactinomycetaceae*		0
Firmicutes	*Clostridia*	*Clostridiales*	*[Tissierellaceae]*	*Gallicola*	0.006666667
Firmicutes	*Bacilli*	*Lactobacillales*	*Enterococcaceae*		0.006666667
Firmicutes	*Bacilli*	*Lactobacillales*	*Aerococcaceae*	*Alkalibacterium*	0.08
Firmicutes	*Erysipelotrichi*	*Erysipelotrichales*	*Erysipelotrichaceae*	*[Eubacterium]*	0.02
Firmicutes	*Bacilli*	*Lactobacillales*			0
Firmicutes	*Bacilli*	*Lactobacillales*	*Leuconostocaceae*		0
Firmicutes	*Bacilli*	*Bacillales*	*Planococcaceae*	*Solibacillus*	0.033333333
Firmicutes	*Clostridia*	*Clostridiales*	*[Tissierellaceae]*	*Anaerococcus*	0
Firmicutes	*Clostridia*	*Clostridiales*	*Veillonellaceae*	*Veillonella*	0
Firmicutes	*Clostridia*	*Clostridiales*	*Veillonellaceae*	*Selenomonas*	0.02
Firmicutes	*Clostridia*	*Clostridiales*	*Ruminococcaceae*	*Faecalibacterium*	0
Firmicutes	*Erysipelotrichi*	*Erysipelotrichales*	*Erysipelotrichaceae*	*L7A_E11*	0
Firmicutes	*Bacilli*	*Lactobacillales*	*Aerococcaceae*	*Aerococcus*	0.093333333
Firmicutes	*Bacilli*	*Bacillales*	*Alicyclobacillaceae*	*Alicyclobacillus*	0.06

Firmicutes	*Clostridia*	*Clostridiales*	*[Tissierellaceae]*	*Peptoniphilus*	0.013333333
Firmicutes	*Erysipelotrichi*	*Erysipelotrichales*	*Erysipelotrichaceae*	*Coprobacillus*	0.026666667
Firmicutes	*Clostridia*	*Clostridiales*	*Veillonellaceae*	*Phascolarctobacterium*	0.02
Firmicutes	*Clostridia*	*Clostridiales*	*Veillonellaceae*	*Anaerovibrio*	0.013333333
Firmicutes	*Bacilli*	*Lactobacillales*	*Carnobacteriaceae*	*Carnobacterium*	0
Firmicutes	*Bacilli*	*Bacillales*	*Planococcaceae*	*Planococcus*	0.046666667
Firmicutes	*Clostridia*	*Clostridiales*	*[Acidaminobacteraceae]*	*Guggenheimella*	0.026666667
Firmicutes	*Clostridia*	*Clostridiales*	*[Tissierellaceae]*	*Parvimonas*	0
Firmicutes	*Bacilli*	*Lactobacillales*	*Carnobacteriaceae*		0.026666667
Firmicutes	*Bacilli*	*Gemellales*	*Gemellaceae*		0
Firmicutes	*Erysipelotrichi*	*Erysipelotrichales*	*Erysipelotrichaceae*	*p-75-a5*	0.006666667
Firmicutes	*Clostridia*	*Clostridiales*	*Caldicoprobacteraceae*	*Caldicoprobacter*	0
Firmicutes	*Erysipelotrichi*	*Erysipelotrichales*	*Erysipelotrichaceae*	*Allobaculum*	0
Firmicutes	*Clostridia*	*Clostridiales*	*Peptococcaceae*	*Peptococcus*	0
Actinobacteria	*Acidimicrobiia*	*Acidimicrobiales*			0.033333333
Actinobacteria	*Actinobacteria*	*Actinomycetales*	*Nocardiopsaceae*		0.02
Actinobacteria	*Actinobacteria*	*Actinomycetales*	*Corynebacteriaceae*	*Corynebacterium*	3.7
Actinobacteria	*Actinobacteria*	*Actinomycetales*	*Yaniellaceae*	*Yaniella*	0.486666667
Actinobacteria	*Actinobacteria*	*Actinomycetales*	*Promicromonosporaceae*		0
Actinobacteria	*Actinobacteria*	*Bifidobacteriales*	*Bifidobacteriaceae*		0.006666667
Actinobacteria	*Actinobacteria*	*Actinomycetales*	*Nocardiopsaceae*	*Thermobifida*	0.006666667
Actinobacteria	*Acidimicrobiia*	*Acidimicrobiales*	*AKIW874*		0
Actinobacteria	*Actinobacteria*	*Actinomycetales*	*Micrococcaceae*		0.306666667
Actinobacteria	*Actinobacteria*	*Actinomycetales*	*Micrococcaceae*	*Arthrobacter*	0.046666667
Actinobacteria	*Actinobacteria*	*Actinomycetales*	*Dermabacteraceae*		0.053333333
Actinobacteria	*Actinobacteria*	*Actinomycetales*	*Actinomycetaceae*	*Actinomyces*	0
Actinobacteria	*Actinobacteria*	*Actinomycetales*			0.12

Actinobacteria	*Actinobacteria*	*Actinomycetales*	*Intrasporangiaceae*		0.48
Actinobacteria	*Actinobacteria*	*Actinomycetales*	*Nocardiopsaceae*	*Prauseria*	0.02
Actinobacteria	*Actinobacteria*	*Actinomycetales*	*Promicromonosporaceae*	*Promicromonospora*	0
Actinobacteria	*Actinobacteria*	*Actinomycetales*	*Promicromonosporaceae*	*Xylanimicrobium*	0.013333333
Actinobacteria	*Actinobacteria*	*Actinomycetales*	*Bogoriellaceae*	*Georgenia*	0.073333333
Actinobacteria	*Actinobacteria*	*Actinomycetales*	*Cellulomonadaceae*	*Oerskovia*	0
Actinobacteria	*Actinobacteria*	*Bifidobacteriales*	*Bifidobacteriaceae*	*Bifidobacterium*	0.533333333
Actinobacteria	*Actinobacteria*	*Actinomycetales*	*Micrococcaceae*	*Kocuria*	0.253333333
Actinobacteria	*Actinobacteria*	*Actinomycetales*	*Dermacoccaceae*	*Dermacoccus*	0.04
Actinobacteria	*Actinobacteria*	*Actinomycetales*	*Micrococcaceae*	*Micrococcus*	0.273333333
Actinobacteria	*Actinobacteria*	*Actinomycetales*	*Micrococcaceae*	*Rothia*	0
Actinobacteria	*Actinobacteria*	*Actinomycetales*	*Brevibacteriaceae*	*Brevibacterium*	0.086666667
Actinobacteria	*Actinobacteria*	*Actinomycetales*	*Actinomycetaceae*	*N09*	0
Actinobacteria	*Actinobacteria*	*Actinomycetales*	*Pseudonocardiaceae*	*Saccharopolyspora*	0
Actinobacteria	*Actinobacteria*	*Actinomycetales*	*Actinomycetaceae*		0.033333333
Actinobacteria	*Actinobacteria*	*Actinomycetales*	*Dermabacteraceae*	*Brachybacterium*	0.16
Actinobacteria	*Actinobacteria*	*Actinomycetales*	*Micrococcaceae*	*Nesterenkonia*	0.14
Actinobacteria	*Actinobacteria*	*Actinomycetales*	*Nocardioidaceae*		0.046666667
Actinobacteria	*Actinobacteria*	*Actinomycetales*	*Microbacteriaceae*		0.006666667
Actinobacteria	*Actinobacteria*	*Actinomycetales*	*Micromonosporaceae*		0
Actinobacteria	*Actinobacteria*	*Actinomycetales*	*Micrococcaceae*	*Microbispora*	0.013333333
Actinobacteria	*Actinobacteria*	*Actinomycetales*	*Pseudonocardiaceae*	*Saccharomonospora*	0.006666667
Actinobacteria	*Actinobacteria*	*Actinomycetales*	*Dermatophilaceae*	*Dermatophilus*	0.013333333
Actinobacteria	*Actinobacteria*	*Actinomycetales*	*Dietziaceae*		0.006666667
Actinobacteria	*Actinobacteria*	*Actinomycetales*	*Dietziaceae*	*Dietzia*	0
Actinobacteria	*Actinobacteria*	*Actinomycetales*	*Microbacteriaceae*	*Microbacterium*	0
Actinobacteria	*Actinobacteria*	*Actinomycetales*	*Mycobacteriaceae*	*Mycobacterium*	0

Actinobacteria	*Coriobacteriia*	*Coriobacteriales*	*Coriobacteriaceae*		0.033333333
Actinobacteria	*Actinobacteria*	*Actinomycetales*	*Gordoniaceae*	*Gordonia*	0.246666667
Actinobacteria	*Actinobacteria*	*Actinomycetales*	*Nocardiaceae*	*Rhodococcus*	0
Actinobacteria	*Actinobacteria*	*Actinomycetales*	*Microbacteriaceae*	*Rathayibacter*	0
Actinobacteria	*Actinobacteria*	*Actinomycetales*	*Pseudonocardiaceae*		0.006666667
Actinobacteria	*Coriobacteriia*	*Coriobacteriales*	*Coriobacteriaceae*	*Atopobium*	0.006666667
Actinobacteria	*Actinobacteria*	*Actinomycetales*	*Streptomycetaceae*	*Streptomyces*	0.013333333
Actinobacteria	*Actinobacteria*	*Actinomycetales*	*Microbacteriaceae*	*Leucobacter*	0.013333333
Actinobacteria	*Actinobacteria*	*Actinomycetales*	*Actinomycetaceae*	*Trueperella*	0.073333333
Actinobacteria	*Actinobacteria*	*Actinomycetales*	*Glycomycetaceae*	*Glycomyces*	0.006666667
Actinobacteria	*Actinobacteria*	*Actinomycetales*	*Sanguibacteraceae*	*Sanguibacter*	0
Actinobacteria	*Actinobacteria*	*Actinomycetales*	*Microbacteriaceae*	*Pseudoclavibacter*	0
Actinobacteria	*Actinobacteria*	*Actinomycetales*	*Cellulomonadaceae*	*Demequina*	0
Actinobacteria	*Actinobacteria*	*Actinomycetales*	*Micromonosporaceae*	*Catellatospora*	0
Bacteroidetes	*Bacteroidia*	*Bacteroidales*	*Rikenellaceae*		0.386666667
Bacteroidetes	*Bacteroidia*	*Bacteroidales*	*Porphyromonadaceae*		0.093333333
Bacteroidetes	*Bacteroidia*	*Bacteroidales*	*Porphyromonadaceae*	*Porphyromonas*	0.006666667
Bacteroidetes	*Bacteroidia*	*Bacteroidales*			0.86
Bacteroidetes	*Bacteroidia*	*Bacteroidales*	*Bacteroidaceae*	*5-7N15*	0.806666667
Bacteroidetes	*Flavobacteriia*	*Flavobacteriales*	*[Weeksellaceae]*		0.006666667
Bacteroidetes	*Sphingobacteriia*	*Sphingobacteriales*	*Sphingobacteriaceae*		0.006666667
Bacteroidetes	*Bacteroidia*	*Bacteroidales*	*RF16*		0.333333333
Bacteroidetes	*Bacteroidia*	*Bacteroidales*	*S24-7*		0.08
Bacteroidetes	*Bacteroidia*	*Bacteroidales*	*ML635J-40*		0
Bacteroidetes	*Bacteroidia*	*Bacteroidales*	*[Paraprevotellaceae]*	*CF231*	0.113333333
Bacteroidetes	*Bacteroidia*	*Bacteroidales*	*SB-1*		0.006666667
Bacteroidetes	*Flavobacteriia*	*Flavobacteriales*	*[Weeksellaceae]*	*Cloacibacterium*	0.006666667

Bacteroidetes	*Bacteroidia*	*Bacteroidales*	*BS11*		0
Bacteroidetes	*Flavobacteriia*	*Flavobacteriales*	*[Weeksellaceae]*	*Chryseobacterium*	0
Bacteroidetes	*Bacteroidia*	*Bacteroidales*	*Prevotellaceae*	*Prevotella*	0.02
Bacteroidetes	*Bacteroidia*	*Bacteroidales*	*Bacteroidaceae*		0.06
Bacteroidetes	*Flavobacteriia*	*Flavobacteriales*	*Flavobacteriaceae*		0.033333333
Bacteroidetes	*Bacteroidia*	*Bacteroidales*	*p-2534-18B5*		0.013333333
Bacteroidetes	*Bacteroidia*	*Bacteroidales*	*Porphyromonadaceae*	*Paludibacter*	0.08
Bacteroidetes	*Bacteroidia*	*Bacteroidales*	*Bacteroidaceae*	*Bacteroides*	0.006666667
Bacteroidetes	*Bacteroidia*	*Bacteroidales*	*[Paraprevotellaceae]*	*[Prevotella]*	0.026666667
Bacteroidetes	*Bacteroidia*	*Bacteroidales*	*Marinilabiaceae*		0.026666667
Bacteroidetes	*[Saprospirae]*	*[Saprospirales]*	*Saprospiraceae*		0
Bacteroidetes	*Bacteroidia*	*Bacteroidales*	*[Paraprevotellaceae]*	*YRC22*	0.013333333
Bacteroidetes	*Flavobacteriia*	*Flavobacteriales*	*[Weeksellaceae]*	*Riemerella*	0
Bacteroidetes	*Sphingobacteriia*	*Sphingobacteriales*	*Sphingobacteriaceae*	*Pedobacter*	0
Bacteroidetes	*Bacteroidia*	*Bacteroidales*	*Porphyromonadaceae*	*Parabacteroides*	0
Bacteroidetes	*[Rhodothermi]*	*[Rhodothermales]*	*Rhodothermaceae*		0
Chloroflexi	*Thermomicrobia*	*JG30-KF-CM45*			0.02
Chloroflexi	*Anaerolineae*	*GCA004*			0
Chloroflexi	*Anaerolineae*	*CFB-26*			0
Chloroflexi	*Anaerolineae*	*Anaerolineales*	*Anaerolinaceae*	*T78*	0.013333333
Cyanobacteria	*4C0d-2*	*YS2*			0
Deferribacteres	*Deferribacteres*	*Deferribacterales*	*Deferribacteraceae*	*Mucispirillum*	0
Fibrobacteres	*TG3*	*TG3-1*	*TSCOR003-O20*		0
Fibrobacteres	*Fibrobacteria*	*Fibrobacterales*	*Fibrobacteraceae*	*Fibrobacter*	0
Fusobacteria	*Fusobacteriia*	*Fusobacteriales*	*Leptotrichiaceae*	*Leptotrichia*	0
Fusobacteria	*Fusobacteriia*	*Fusobacteriales*	*Fusobacteriaceae*	*Fusobacterium*	0.126666667
Fusobacteria	*Fusobacteriia*	*Fusobacteriales*			0

Fusobacteria	*Fusobacteriia*	*Fusobacteriales*	*Leptotrichiaceae*	*Sneathia*	0.113333333
Gemmatimonadetes	*Gemm-5*				0
Proteobacteria	*Gammaproteobacteria*	*Pseudomonadales*	*Moraxellaceae*	*Acinetobacter*	1.186666667
Proteobacteria	*Gammaproteobacteria*	*Pseudomonadales*	*Moraxellaceae*	*Psychrobacter*	0.086666667
Proteobacteria	*Gammaproteobacteria*	*Pasteurellales*	*Pasteurellaceae*	*Haemophilus*	0.006666667
Proteobacteria	*Gammaproteobacteria*	*Methylococcales*	*Methylococcaceae*	*Methylocaldum*	0.006666667
Proteobacteria	*Gammaproteobacteria*	*Thiotrichales*	*Thiotrichaceae*	*Thiothrix*	0
Proteobacteria	*Betaproteobacteria*	*Rhodocyclales*	*Rhodocyclaceae*	*Thauera*	0
Proteobacteria	*Gammaproteobacteria*	*Pasteurellales*	*Pasteurellaceae*	*Aggregatibacter*	0
Proteobacteria	*Gammaproteobacteria*	*Pseudomonadales*	*Pseudomonadaceae*		0.073333333
Proteobacteria	*Alphaproteobacteria*	*Rhodobacterales*	*Rhodobacteraceae*		0.013333333
Proteobacteria	*Gammaproteobacteria*	*Legionellales*	*Legionellaceae*	*Legionella*	0
Proteobacteria	*Alphaproteobacteria*	*Rhodobacterales*	*Rhodobacteraceae*	*Paracoccus*	0.133333333
Proteobacteria	*Alphaproteobacteria*	*Rhizobiales*	*Phyllobacteriaceae*		0.006666667
Proteobacteria	*Gammaproteobacteria*	*Enterobacteriales*	*Enterobacteriaceae*	*Citrobacter*	0.02
Proteobacteria	*Gammaproteobacteria*	*Thiotrichales*	*Piscirickettsiaceae*		0.013333333
Proteobacteria	*Gammaproteobacteria*	*Pseudomonadales*	*Pseudomonadaceae*	*Pseudomonas*	0.453333333
Proteobacteria	*Gammaproteobacteria*	*Alteromonadales*	*Alteromonadaceae*	*BD2-13*	0
Proteobacteria	*Alphaproteobacteria*	*Rhizobiales*	*Rhizobiaceae*	*Agrobacterium*	0.006666667
Proteobacteria	*Betaproteobacteria*	*Burkholderiales*	*Burkholderiaceae*	*Burkholderia*	0
Proteobacteria	*Alphaproteobacteria*	*Rhizobiales*	*Hyphomicrobiaceae*	*Devosia*	0
Proteobacteria	*Betaproteobacteria*	*Burkholderiales*	*Alcaligenaceae*		0.04
Proteobacteria	*Betaproteobacteria*	*Burkholderiales*	*Burkholderiaceae*	*Lautropia*	0
Proteobacteria	*Betaproteobacteria*	*Burkholderiales*	*Alcaligenaceae*	*Oligella*	0
Proteobacteria	*Gammaproteobacteria*	*Oceanospirillales*	*Halomonadaceae*	*Halomonas*	0.12
Proteobacteria	*Alphaproteobacteria*	*Rhizobiales*	*Bradyrhizobiaceae*		0.066666667
Proteobacteria	*Betaproteobacteria*	*Burkholderiales*	*Comamonadaceae*		0.026666667

Proteobacteria	*Epsilonproteobacteria*	*Campylobacterales*	*Campylobacteraceae*	*Campylobacter*	0
Proteobacteria	*Alphaproteobacteria*	*Sphingomonadales*	*Sphingomonadaceae*	*Novosphingobium*	0
Proteobacteria	*Gammaproteobacteria*	*Legionellales*	*Legionellaceae*		0
Proteobacteria	*Epsilonproteobacteria*	*Campylobacterales*	*Campylobacteraceae*	*Arcobacter*	0.006666667
Proteobacteria	*Betaproteobacteria*	*Burkholderiales*	*Oxalobacteraceae*		0.006666667
Proteobacteria	*Gammaproteobacteria*	*Enterobacteriales*	*Enterobacteriaceae*		0.4
Proteobacteria	*Alphaproteobacteria*	*Sphingomonadales*	*Sphingomonadaceae*	*Sphingomonas*	0.006666667
Proteobacteria	*Gammaproteobacteria*	*Enterobacteriales*	*Enterobacteriaceae*	*Providencia*	0
Proteobacteria	*Gammaproteobacteria*	*Pasteurellales*	*Pasteurellaceae*	*Pasteurella*	0.113333333
Proteobacteria	*Gammaproteobacteria*	*Aeromonadales*	*Succinivibrionaceae*	*Ruminobacter*	0.14
Proteobacteria	*Gammaproteobacteria*	*Xanthomonadales*	*Xanthomonadaceae*	*Pseudoxanthomonas*	0
Proteobacteria	*Alphaproteobacteria*	*Sphingomonadales*	*Erythrobacteraceae*		0
Proteobacteria	*Gammaproteobacteria*	*Pseudomonadales*	*Moraxellaceae*	*Moraxella*	0
Proteobacteria	*Deltaproteobacteria*	*Desulfovibrionales*	*Desulfovibrionaceae*		0.033333333
Proteobacteria	*Betaproteobacteria*	*Rhodocyclales*	*Rhodocyclaceae*	*Hydrogenophilus*	0
Proteobacteria	*Betaproteobacteria*	*Neisseriales*	*Neisseriaceae*	*Neisseria*	0.006666667
Proteobacteria	*Gammaproteobacteria*	*Enterobacteriales*	*Enterobacteriaceae*	*Erwinia*	0
Proteobacteria	*Gammaproteobacteria*	*Enterobacteriales*	*Enterobacteriaceae*	*Proteus*	0
Proteobacteria	*Betaproteobacteria*	*Rhodocyclales*	*Rhodocyclaceae*	*Dechloromonas*	0
Proteobacteria	*Gammaproteobacteria*	*Chromatiales*	*Chromatiaceae*	*Allochromatium*	0
Proteobacteria	*Alphaproteobacteria*				0.02
Proteobacteria	*Gammaproteobacteria*	*Xanthomonadales*	*Xanthomonadaceae*	*Luteimonas*	0.053333333
Proteobacteria	*Gammaproteobacteria*				0.006666667
Proteobacteria	*Gammaproteobacteria*	*Aeromonadales*	*Succinivibrionaceae*	*Succinivibrio*	0.113333333
Proteobacteria	*Alphaproteobacteria*	*Rhizobiales*	*Hyphomicrobiaceae*	*Hyphomicrobium*	0
Proteobacteria	*Gammaproteobacteria*	*Alteromonadales*	*Alteromonadaceae*	*Marinobacter*	0.1
Proteobacteria	*Gammaproteobacteria*	*Pasteurellales*	*Pasteurellaceae*	*Actinobacillus*	0

Proteobacteria	*Gammaproteobacteria*	*Oceanospirillales*	*Oceanospirillaceae*		0
Proteobacteria	*Gammaproteobacteria*	*Pseudomonadales*	*Moraxellaceae*		0.026666667
Proteobacteria	*Gammaproteobacteria*	*Vibrionales*	*Pseudoalteromonadaceae*		0
Proteobacteria	*Deltaproteobacteria*	*Myxococcales*			0
Proteobacteria	*Gammaproteobacteria*	*Pseudomonadales*	*Moraxellaceae*	*Enhydrobacter*	0.14
Proteobacteria	*Gammaproteobacteria*	*Pasteurellales*	*Pasteurellaceae*		0
Proteobacteria	*Gammaproteobacteria*	*Enterobacteriales*	*Enterobacteriaceae*	*Serratia*	0
Proteobacteria	*Betaproteobacteria*	*Burkholderiales*	*Oxalobacteraceae*	*Ralstonia*	0
Proteobacteria	*Alphaproteobacteria*	*Sphingomonadales*	*Sphingomonadaceae*		0
Proteobacteria	*Betaproteobacteria*	*Burkholderiales*	*Comamonadaceae*	*Delftia*	0
Proteobacteria	*Betaproteobacteria*	*Burkholderiales*	*Oxalobacteraceae*	*Janthinobacterium*	0
Proteobacteria	*Betaproteobacteria*	*Neisseriales*	*Neisseriaceae*		0.006666667
Proteobacteria	*Gammaproteobacteria*	*Xanthomonadales*	*Xanthomonadaceae*	*Lysobacter*	0
Proteobacteria	*Betaproteobacteria*	*Neisseriales*	*Neisseriaceae*	*Vitreoscilla*	0
Proteobacteria	*Gammaproteobacteria*	*Alteromonadales*	*Idiomarinaceae*		0.006666667
Proteobacteria	*Gammaproteobacteria*	*Aeromonadales*	*Aeromonadaceae*		0
Proteobacteria	*Deltaproteobacteria*	*Desulfovibrionales*	*Desulfomicrobiaceae*	*Desulfomicrobium*	0
Proteobacteria	*Betaproteobacteria*	*Rhodocyclales*	*Rhodocyclaceae*		0.02
Proteobacteria	*Betaproteobacteria*	*Rhodocyclales*	*Rhodocyclaceae*	*Azospira*	0
Proteobacteria	*Gammaproteobacteria*	*Xanthomonadales*	*Xanthomonadaceae*	*Stenotrophomonas*	0.006666667
Proteobacteria	*Gammaproteobacteria*	*Legionellales*	*Coxiellaceae*	*Coxiella*	0.146666667
Proteobacteria	*Alphaproteobacteria*	*Rhizobiales*	*Hyphomicrobiaceae*		0.006666667
Proteobacteria	*Gammaproteobacteria*	*Chromatiales*	*Ectothiorhodospiraceae*		0
Proteobacteria	*Alphaproteobacteria*	*Rhizobiales*	*Methylobacteriaceae*	*Methylobacterium*	0.006666667
Proteobacteria	*Gammaproteobacteria*	*Alteromonadales*			0
Proteobacteria	*Gammaproteobacteria*	*Alteromonadales*	*Alteromonadaceae*	*Marinimicrobium*	0
Proteobacteria	*Alphaproteobacteria*	*Rhizobiales*	*Bradyrhizobiaceae*	*Balneimonas*	0

Proteobacteria	*Gammaproteobacteria*	*Xanthomonadales*	*Xanthomonadaceae*		0
Proteobacteria	*Betaproteobacteria*	*Burkholderiales*	*Comamonadaceae*	*Limnohabitans*	0
Proteobacteria	*Gammaproteobacteria*	*Vibrionales*	*Vibrionaceae*	*Vibrio*	0
Proteobacteria	*Deltaproteobacteria*	*Desulfobacterales*	*Desulfobulbaceae*		0
Proteobacteria	*Gammaproteobacteria*	*Alteromonadales*	*Alteromonadaceae*		0.006666667
Proteobacteria	*Gammaproteobacteria*	*Alteromonadales*	*Alteromonadaceae*	*Candidatus Endobugula*	0.02
Proteobacteria	*Alphaproteobacteria*	*RF32*			0
Proteobacteria	*Gammaproteobacteria*	*Chromatiales*			0
Proteobacteria	*Deltaproteobacteria*	*GMD14H09*			0.006666667
Proteobacteria	*Gammaproteobacteria*	*Oceanospirillales*	*Halomonadaceae*	*Candidatus Portiera*	0.013333333
Spirochaetes	*Spirochaetes*	*Spirochaetales*	*Spirochaetaceae*	*Treponema*	0.166666667
Spirochaetes	*Spirochaetes*	*M2PT2-76*			0
Tenericutes	*Mollicutes*	*RF39*			0.326666667
Tenericutes	*Mollicutes*	*Mycoplasmatales*	*Mycoplasmataceae*	*Mycoplasma*	0.26
Tenericutes	*Mollicutes*	*Anaeroplasmatales*	*Anaeroplasmataceae*	*Anaeroplasma*	0.006666667
Tenericutes	*RF3*	*ML615J-28*			0
Tenericutes	*Mollicutes*	*Anaeroplasmatales*	*Anaeroplasmataceae*		0.006666667
Tenericutes	*Mollicutes*	*Mycoplasmatales*	*Mycoplasmataceae*	*Ureaplasma*	0
Verrucomicrobia	*Verrucomicrobiae*	*Verrucomicrobiales*	*Verrucomicrobiaceae*	*Akkermansia*	0.013333333
Verrucomicrobia	*Verruco-5*	*WCHB1-41*	*RFP12*		0.013333333
[Thermi]	*Deinococci*	*Thermales*	*Thermaceae*	*Meiothermus*	0
[Thermi]	*Deinococci*	*Deinococcales*	*Deinococcaceae*	*Deinococcus*	0.046666667

[a] Blank cells represent “Unidentified” taxonomic levels
